# Search for physics beyond the standard model in events with *τ* leptons, jets, and large transverse momentum imbalance in pp collisions at $\sqrt{s}=7\ \mathrm{TeV}$

**DOI:** 10.1140/epjc/s10052-013-2493-8

**Published:** 2013-07-16

**Authors:** The CMS Collaboration, S. Chatrchyan, V. Khachatryan, A. M. Sirunyan, A. Tumasyan, W. Adam, E. Aguilo, T. Bergauer, M. Dragicevic, J. Erö, C. Fabjan, M. Friedl, R. Frühwirth, V. M. Ghete, J. Hammer, N. Hörmann, J. Hrubec, M. Jeitler, W. Kiesenhofer, V. Knünz, M. Krammer, I. Krätschmer, D. Liko, I. Mikulec, M. Pernicka, B. Rahbaran, C. Rohringer, H. Rohringer, R. Schöfbeck, J. Strauss, A. Taurok, W. Waltenberger, G. Walzel, E. Widl, C.-E. Wulz, V. Mossolov, N. Shumeiko, J. Suarez Gonzalez, M. Bansal, S. Bansal, T. Cornelis, E. A. De Wolf, X. Janssen, S. Luyckx, L. Mucibello, S. Ochesanu, B. Roland, R. Rougny, M. Selvaggi, Z. Staykova, H. Van Haevermaet, P. Van Mechelen, N. Van Remortel, A. Van Spilbeeck, F. Blekman, S. Blyweert, J. D’Hondt, R. Gonzalez Suarez, A. Kalogeropoulos, M. Maes, A. Olbrechts, W. Van Doninck, P. Van Mulders, G. P. Van Onsem, I. Villella, B. Clerbaux, G. De Lentdecker, V. Dero, A. P. R. Gay, T. Hreus, A. Léonard, P. E. Marage, A. Mohammadi, T. Reis, L. Thomas, G. Vander Marcken, C. Vander Velde, P. Vanlaer, J. Wang, V. Adler, K. Beernaert, A. Cimmino, S. Costantini, G. Garcia, M. Grunewald, B. Klein, J. Lellouch, A. Marinov, J. Mccartin, A. A. Ocampo Rios, D. Ryckbosch, N. Strobbe, F. Thyssen, M. Tytgat, P. Verwilligen, S. Walsh, E. Yazgan, N. Zaganidis, S. Basegmez, G. Bruno, R. Castello, L. Ceard, C. Delaere, T. du Pree, D. Favart, L. Forthomme, A. Giammanco, J. Hollar, V. Lemaitre, J. Liao, O. Militaru, C. Nuttens, D. Pagano, A. Pin, K. Piotrzkowski, N. Schul, J. M. Vizan Garcia, N. Beliy, T. Caebergs, E. Daubie, G. H. Hammad, G. A. Alves, M. Correa Martins Junior, D. De Jesus Damiao, T. Martins, M. E. Pol, M. H. G. Souza, W. L. Aldá Júnior, W. Carvalho, A. Custódio, E. M. Da Costa, C. De Oliveira Martins, S. Fonseca De Souza, D. Matos Figueiredo, L. Mundim, H. Nogima, V. Oguri, W. L. Prado Da Silva, A. Santoro, L. Soares Jorge, A. Sznajder, T. S. Anjos, C. A. Bernardes, F. A. Dias, T. R. Fernandez Perez Tomei, E. M. Gregores, C. Lagana, F. Marinho, P. G. Mercadante, S. F. Novaes, Sandra S. Padula, V. Genchev, P. Iaydjiev, S. Piperov, M. Rodozov, S. Stoykova, G. Sultanov, V. Tcholakov, R. Trayanov, M. Vutova, A. Dimitrov, R. Hadjiiska, V. Kozhuharov, L. Litov, B. Pavlov, P. Petkov, J. G. Bian, G. M. Chen, H. S. Chen, C. H. Jiang, D. Liang, S. Liang, X. Meng, J. Tao, J. Wang, X. Wang, Z. Wang, H. Xiao, M. Xu, J. Zang, Z. Zhang, C. Asawatangtrakuldee, Y. Ban, Y. Guo, W. Li, S. Liu, Y. Mao, S. J. Qian, H. Teng, D. Wang, L. Zhang, W. Zou, C. Avila, J. P. Gomez, B. Gomez Moreno, A. F. Osorio Oliveros, J. C. Sanabria, N. Godinovic, D. Lelas, R. Plestina, D. Polic, I. Puljak, Z. Antunovic, M. Kovac, V. Brigljevic, S. Duric, K. Kadija, J. Luetic, S. Morovic, A. Attikis, M. Galanti, G. Mavromanolakis, J. Mousa, C. Nicolaou, F. Ptochos, P. A. Razis, M. Finger, M. Finger, Y. Assran, S. Elgammal, A. Ellithi Kamel, M. A. Mahmoud, A. Radi, M. Kadastik, M. Müntel, M. Raidal, L. Rebane, A. Tiko, P. Eerola, G. Fedi, M. Voutilainen, J. Härkönen, A. Heikkinen, V. Karimäki, R. Kinnunen, M. J. Kortelainen, T. Lampén, K. Lassila-Perini, S. Lehti, T. Lindén, P. Luukka, T. Mäenpää, T. Peltola, E. Tuominen, J. Tuominiemi, E. Tuovinen, D. Ungaro, L. Wendland, K. Banzuzi, A. Karjalainen, A. Korpela, T. Tuuva, M. Besancon, S. Choudhury, M. Dejardin, D. Denegri, B. Fabbro, J. L. Faure, F. Ferri, S. Ganjour, A. Givernaud, P. Gras, G. Hamel de Monchenault, P. Jarry, E. Locci, J. Malcles, L. Millischer, A. Nayak, J. Rander, A. Rosowsky, I. Shreyber, M. Titov, S. Baffioni, F. Beaudette, L. Benhabib, L. Bianchini, M. Bluj, C. Broutin, P. Busson, C. Charlot, N. Daci, T. Dahms, L. Dobrzynski, R. Granier de Cassagnac, M. Haguenauer, P. Miné, C. Mironov, I. N. Naranjo, M. Nguyen, C. Ochando, P. Paganini, D. Sabes, R. Salerno, Y. Sirois, C. Veelken, A. Zabi, J.-L. Agram, J. Andrea, D. Bloch, D. Bodin, J.-M. Brom, M. Cardaci, E. C. Chabert, C. Collard, E. Conte, F. Drouhin, C. Ferro, J.-C. Fontaine, D. Gelé, U. Goerlach, P. Juillot, A.-C. Le Bihan, P. Van Hove, F. Fassi, D. Mercier, S. Beauceron, N. Beaupere, O. Bondu, G. Boudoul, J. Chasserat, R. Chierici, D. Contardo, P. Depasse, H. El Mamouni, J. Fay, S. Gascon, M. Gouzevitch, B. Ille, T. Kurca, M. Lethuillier, L. Mirabito, S. Perries, L. Sgandurra, V. Sordini, Y. Tschudi, P. Verdier, S. Viret, Z. Tsamalaidze, G. Anagnostou, C. Autermann, S. Beranek, M. Edelhoff, L. Feld, N. Heracleous, O. Hindrichs, R. Jussen, K. Klein, J. Merz, A. Ostapchuk, A. Perieanu, F. Raupach, J. Sammet, S. Schael, D. Sprenger, H. Weber, B. Wittmer, V. Zhukov, M. Ata, J. Caudron, E. Dietz-Laursonn, D. Duchardt, M. Erdmann, R. Fischer, A. Güth, T. Hebbeker, C. Heidemann, K. Hoepfner, D. Klingebiel, P. Kreuzer, M. Merschmeyer, A. Meyer, M. Olschewski, P. Papacz, H. Pieta, H. Reithler, S. A. Schmitz, L. Sonnenschein, J. Steggemann, D. Teyssier, M. Weber, M. Bontenackels, V. Cherepanov, Y. Erdogan, G. Flügge, H. Geenen, M. Geisler, W. Haj Ahmad, F. Hoehle, B. Kargoll, T. Kress, Y. Kuessel, J. Lingemann, A. Nowack, L. Perchalla, O. Pooth, P. Sauerland, A. Stahl, M. Aldaya Martin, J. Behr, W. Behrenhoff, U. Behrens, M. Bergholz, A. Bethani, K. Borras, A. Burgmeier, A. Cakir, L. Calligaris, A. Campbell, E. Castro, F. Costanza, D. Dammann, C. Diez Pardos, G. Eckerlin, D. Eckstein, G. Flucke, A. Geiser, I. Glushkov, P. Gunnellini, S. Habib, J. Hauk, G. Hellwig, H. Jung, M. Kasemann, P. Katsas, C. Kleinwort, H. Kluge, A. Knutsson, M. Krämer, D. Krücker, E. Kuznetsova, W. Lange, W. Lohmann, B. Lutz, R. Mankel, I. Marfin, M. Marienfeld, I.-A. Melzer-Pellmann, A. B. Meyer, J. Mnich, A. Mussgiller, S. Naumann-Emme, O. Novgorodova, J. Olzem, H. Perrey, A. Petrukhin, D. Pitzl, A. Raspereza, P. M. Ribeiro Cipriano, C. Riedl, E. Ron, M. Rosin, J. Salfeld-Nebgen, R. Schmidt, T. Schoerner-Sadenius, N. Sen, A. Spiridonov, M. Stein, R. Walsh, C. Wissing, V. Blobel, J. Draeger, H. Enderle, J. Erfle, U. Gebbert, M. Görner, T. Hermanns, R. S. Höing, K. Kaschube, G. Kaussen, H. Kirschenmann, R. Klanner, J. Lange, B. Mura, F. Nowak, T. Peiffer, N. Pietsch, D. Rathjens, C. Sander, H. Schettler, P. Schleper, E. Schlieckau, A. Schmidt, M. Schröder, T. Schum, M. Seidel, V. Sola, H. Stadie, G. Steinbrück, J. Thomsen, L. Vanelderen, C. Barth, J. Berger, C. Böser, T. Chwalek, W. De Boer, A. Descroix, A. Dierlamm, M. Feindt, M. Guthoff, C. Hackstein, F. Hartmann, T. Hauth, M. Heinrich, H. Held, K. H. Hoffmann, S. Honc, I. Katkov, J. R. Komaragiri, P. Lobelle Pardo, D. Martschei, S. Mueller, Th. Müller, M. Niegel, A. Nürnberg, O. Oberst, A. Oehler, J. Ott, G. Quast, K. Rabbertz, F. Ratnikov, N. Ratnikova, S. Röcker, A. Scheurer, F.-P. Schilling, G. Schott, H. J. Simonis, F. M. Stober, D. Troendle, R. Ulrich, J. Wagner-Kuhr, S. Wayand, T. Weiler, M. Zeise, G. Daskalakis, T. Geralis, S. Kesisoglou, A. Kyriakis, D. Loukas, I. Manolakos, A. Markou, C. Markou, C. Mavrommatis, E. Ntomari, L. Gouskos, T. J. Mertzimekis, A. Panagiotou, N. Saoulidou, I. Evangelou, C. Foudas, P. Kokkas, N. Manthos, I. Papadopoulos, V. Patras, G. Bencze, C. Hajdu, P. Hidas, D. Horvath, F. Sikler, V. Veszpremi, G. Vesztergombi, N. Beni, S. Czellar, J. Molnar, J. Palinkas, Z. Szillasi, J. Karancsi, P. Raics, Z. L. Trocsanyi, B. Ujvari, S. B. Beri, V. Bhatnagar, N. Dhingra, R. Gupta, M. Kaur, M. Z. Mehta, N. Nishu, L. K. Saini, A. Sharma, J. B. Singh, Ashok Kumar, Arun Kumar, S. Ahuja, A. Bhardwaj, B. C. Choudhary, S. Malhotra, M. Naimuddin, K. Ranjan, V. Sharma, R. K. Shivpuri, S. Banerjee, S. Bhattacharya, S. Dutta, B. Gomber, Sa. Jain, Sh. Jain, R. Khurana, S. Sarkar, M. Sharan, A. Abdulsalam, R. K. Choudhury, D. Dutta, S. Kailas, V. Kumar, P. Mehta, A. K. Mohanty, L. M. Pant, P. Shukla, T. Aziz, S. Ganguly, M. Guchait, M. Maity, G. Majumder, K. Mazumdar, G. B. Mohanty, B. Parida, K. Sudhakar, N. Wickramage, S. Banerjee, S. Dugad, H. Arfaei, H. Bakhshiansohi, S. M. Etesami, A. Fahim, M. Hashemi, H. Hesari, A. Jafari, M. Khakzad, M. Mohammadi Najafabadi, S. Paktinat Mehdiabadi, B. Safarzadeh, M. Zeinali, M. Abbrescia, L. Barbone, C. Calabria, S. S. Chhibra, A. Colaleo, D. Creanza, N. De Filippis, M. De Palma, L. Fiore, G. Iaselli, L. Lusito, G. Maggi, M. Maggi, B. Marangelli, S. My, S. Nuzzo, N. Pacifico, A. Pompili, G. Pugliese, G. Selvaggi, L. Silvestris, G. Singh, R. Venditti, G. Zito, G. Abbiendi, A. C. Benvenuti, D. Bonacorsi, S. Braibant-Giacomelli, L. Brigliadori, P. Capiluppi, A. Castro, F. R. Cavallo, M. Cuffiani, G. M. Dallavalle, F. Fabbri, A. Fanfani, D. Fasanella, P. Giacomelli, C. Grandi, L. Guiducci, S. Marcellini, G. Masetti, M. Meneghelli, A. Montanari, F. L. Navarria, F. Odorici, A. Perrotta, F. Primavera, A. M. Rossi, T. Rovelli, G. P. Siroli, R. Travaglini, S. Albergo, G. Cappello, M. Chiorboli, S. Costa, R. Potenza, A. Tricomi, C. Tuve, G. Barbagli, V. Ciulli, C. Civinini, R. D’Alessandro, E. Focardi, S. Frosali, E. Gallo, S. Gonzi, M. Meschini, S. Paoletti, G. Sguazzoni, A. Tropiano, L. Benussi, S. Bianco, S. Colafranceschi, F. Fabbri, D. Piccolo, P. Fabbricatore, R. Musenich, S. Tosi, A. Benaglia, F. De Guio, L. Di Matteo, S. Fiorendi, S. Gennai, A. Ghezzi, S. Malvezzi, R. A. Manzoni, A. Martelli, A. Massironi, D. Menasce, L. Moroni, M. Paganoni, D. Pedrini, S. Ragazzi, N. Redaelli, S. Sala, T. Tabarelli de Fatis, S. Buontempo, C. A. Carrillo Montoya, N. Cavallo, A. De Cosa, O. Dogangun, F. Fabozzi, A. O. M. Iorio, L. Lista, S. Meola, M. Merola, P. Paolucci, P. Azzi, N. Bacchetta, D. Bisello, A. Branca, R. Carlin, P. Checchia, T. Dorigo, F. Gasparini, U. Gasparini, A. Gozzelino, K. Kanishchev, S. Lacaprara, I. Lazzizzera, M. Margoni, A. T. Meneguzzo, J. Pazzini, N. Pozzobon, P. Ronchese, F. Simonetto, E. Torassa, M. Tosi, S. Vanini, P. Zotto, A. Zucchetta, G. Zumerle, M. Gabusi, S. P. Ratti, C. Riccardi, P. Torre, P. Vitulo, M. Biasini, G. M. Bilei, L. Fanò, P. Lariccia, A. Lucaroni, G. Mantovani, M. Menichelli, A. Nappi, F. Romeo, A. Saha, A. Santocchia, A. Spiezia, S. Taroni, P. Azzurri, G. Bagliesi, J. Bernardini, T. Boccali, G. Broccolo, R. Castaldi, R. T. D’Agnolo, R. Dell’Orso, F. Fiori, L. Foà, A. Giassi, A. Kraan, F. Ligabue, T. Lomtadze, L. Martini, A. Messineo, F. Palla, A. Rizzi, A. T. Serban, P. Spagnolo, P. Squillacioti, R. Tenchini, G. Tonelli, A. Venturi, P. G. Verdini, L. Barone, F. Cavallari, D. Del Re, M. Diemoz, C. Fanelli, M. Grassi, E. Longo, P. Meridiani, F. Micheli, S. Nourbakhsh, G. Organtini, R. Paramatti, S. Rahatlou, M. Sigamani, L. Soffi, N. Amapane, R. Arcidiacono, S. Argiro, M. Arneodo, C. Biino, N. Cartiglia, M. Costa, G. Dellacasa, N. Demaria, C. Mariotti, S. Maselli, E. Migliore, V. Monaco, M. Musich, M. M. Obertino, N. Pastrone, M. Pelliccioni, A. Potenza, A. Romero, R. Sacchi, A. Solano, A. Staiano, A. Vilela Pereira, S. Belforte, V. Candelise, M. Casarsa, F. Cossutti, G. Della Ricca, B. Gobbo, M. Marone, D. Montanino, A. Penzo, A. Schizzi, S. G. Heo, T. Y. Kim, S. K. Nam, S. Chang, D. H. Kim, G. N. Kim, D. J. Kong, H. Park, S. R. Ro, D. C. Son, T. Son, J. Y. Kim, Zero J. Kim, S. Song, S. Choi, D. Gyun, B. Hong, M. Jo, H. Kim, T. J. Kim, K. S. Lee, D. H. Moon, S. K. Park, M. Choi, J. H. Kim, C. Park, I. C. Park, S. Park, G. Ryu, Y. Cho, Y. Choi, Y. K. Choi, J. Goh, M. S. Kim, E. Kwon, B. Lee, J. Lee, S. Lee, H. Seo, I. Yu, M. J. Bilinskas, I. Grigelionis, M. Janulis, A. Juodagalvis, H. Castilla-Valdez, E. De La Cruz-Burelo, I. Heredia-de La Cruz, R. Lopez-Fernandez, R. Magaña Villalba, J. Martínez-Ortega, A. Sanchez-Hernandez, L. M. Villasenor-Cendejas, S. Carrillo Moreno, F. Vazquez Valencia, H. A. Salazar Ibarguen, E. Casimiro Linares, A. Morelos Pineda, M. A. Reyes-Santos, D. Krofcheck, A. J. Bell, P. H. Butler, R. Doesburg, S. Reucroft, H. Silverwood, M. Ahmad, M. H. Ansari, M. I. Asghar, H. R. Hoorani, S. Khalid, W. A. Khan, T. Khurshid, S. Qazi, M. A. Shah, M. Shoaib, H. Bialkowska, B. Boimska, T. Frueboes, R. Gokieli, M. Górski, M. Kazana, K. Nawrocki, K. Romanowska-Rybinska, M. Szleper, G. Wrochna, P. Zalewski, G. Brona, K. Bunkowski, M. Cwiok, W. Dominik, K. Doroba, A. Kalinowski, M. Konecki, J. Krolikowski, N. Almeida, P. Bargassa, A. David, P. Faccioli, P. G. Ferreira Parracho, M. Gallinaro, J. Seixas, J. Varela, P. Vischia, P. Bunin, M. Gavrilenko, I. Golutvin, I. Gorbunov, V. Karjavin, V. Konoplyanikov, G. Kozlov, A. Lanev, A. Malakhov, P. Moisenz, V. Palichik, V. Perelygin, M. Savina, S. Shmatov, V. Smirnov, A. Volodko, A. Zarubin, S. Evstyukhin, V. Golovtsov, Y. Ivanov, V. Kim, P. Levchenko, V. Murzin, V. Oreshkin, I. Smirnov, V. Sulimov, L. Uvarov, S. Vavilov, A. Vorobyev, An. Vorobyev, Yu. Andreev, A. Dermenev, S. Gninenko, N. Golubev, M. Kirsanov, N. Krasnikov, V. Matveev, A. Pashenkov, D. Tlisov, A. Toropin, V. Epshteyn, M. Erofeeva, V. Gavrilov, M. Kossov, N. Lychkovskaya, V. Popov, G. Safronov, S. Semenov, V. Stolin, E. Vlasov, A. Zhokin, V. Andreev, M. Azarkin, I. Dremin, M. Kirakosyan, A. Leonidov, G. Mesyats, S. V. Rusakov, A. Vinogradov, A. Belyaev, E. Boos, V. Bunichev, M. Dubinin, L. Dudko, A. Ershov, A. Gribushin, V. Klyukhin, O. Kodolova, I. Lokhtin, A. Markina, S. Obraztsov, M. Perfilov, S. Petrushanko, A. Popov, L. Sarycheva, V. Savrin, I. Azhgirey, I. Bayshev, S. Bitioukov, V. Grishin, V. Kachanov, D. Konstantinov, V. Krychkine, V. Petrov, R. Ryutin, A. Sobol, L. Tourtchanovitch, S. Troshin, N. Tyurin, A. Uzunian, A. Volkov, P. Adzic, M. Djordjevic, M. Ekmedzic, D. Krpic, J. Milosevic, M. Aguilar-Benitez, J. Alcaraz Maestre, P. Arce, C. Battilana, E. Calvo, M. Cerrada, M. Chamizo Llatas, N. Colino, B. De La Cruz, A. Delgado Peris, D. Domínguez Vázquez, C. Fernandez Bedoya, J. P. Fernández Ramos, A. Ferrando, J. Flix, M. C. Fouz, P. Garcia-Abia, O. Gonzalez Lopez, S. Goy Lopez, J. M. Hernandez, M. I. Josa, G. Merino, J. Puerta Pelayo, A. Quintario Olmeda, I. Redondo, L. Romero, J. Santaolalla, M. S. Soares, C. Willmott, C. Albajar, G. Codispoti, J. F. de Trocóniz, H. Brun, J. Cuevas, J. Fernandez Menendez, S. Folgueras, I. Gonzalez Caballero, L. Lloret Iglesias, J. Piedra Gomez, J. A. Brochero Cifuentes, I. J. Cabrillo, A. Calderon, S. H. Chuang, J. Duarte Campderros, M. Felcini, M. Fernandez, G. Gomez, J. Gonzalez Sanchez, A. Graziano, C. Jorda, A. Lopez Virto, J. Marco, R. Marco, C. Martinez Rivero, F. Matorras, F. J. Munoz Sanchez, T. Rodrigo, A. Y. Rodríguez-Marrero, A. Ruiz-Jimeno, L. Scodellaro, I. Vila, R. Vilar Cortabitarte, D. Abbaneo, E. Auffray, G. Auzinger, M. Bachtis, P. Baillon, A. H. Ball, D. Barney, J. F. Benitez, C. Bernet, G. Bianchi, P. Bloch, A. Bocci, A. Bonato, C. Botta, H. Breuker, T. Camporesi, G. Cerminara, T. Christiansen, J. A. Coarasa Perez, D. D’Enterria, A. Dabrowski, A. De Roeck, S. Di Guida, M. Dobson, N. Dupont-Sagorin, A. Elliott-Peisert, B. Frisch, W. Funk, G. Georgiou, M. Giffels, D. Gigi, K. Gill, D. Giordano, M. Giunta, F. Glege, R. Gomez-Reino Garrido, P. Govoni, S. Gowdy, R. Guida, M. Hansen, P. Harris, C. Hartl, J. Harvey, B. Hegner, A. Hinzmann, V. Innocente, P. Janot, K. Kaadze, E. Karavakis, K. Kousouris, P. Lecoq, Y.-J. Lee, P. Lenzi, C. Lourenço, N. Magini, T. Mäki, M. Malberti, L. Malgeri, M. Mannelli, L. Masetti, F. Meijers, S. Mersi, E. Meschi, R. Moser, M. U. Mozer, M. Mulders, P. Musella, E. Nesvold, T. Orimoto, L. Orsini, E. Palencia Cortezon, E. Perez, L. Perrozzi, A. Petrilli, A. Pfeiffer, M. Pierini, M. Pimiä, D. Piparo, G. Polese, L. Quertenmont, A. Racz, W. Reece, J. Rodrigues Antunes, G. Rolandi, C. Rovelli, M. Rovere, H. Sakulin, F. Santanastasio, C. Schäfer, C. Schwick, I. Segoni, S. Sekmen, A. Sharma, P. Siegrist, P. Silva, M. Simon, P. Sphicas, D. Spiga, A. Tsirou, G. I. Veres, J. R. Vlimant, H. K. Wöhri, S. D. Worm, W. D. Zeuner, W. Bertl, K. Deiters, W. Erdmann, K. Gabathuler, R. Horisberger, Q. Ingram, H. C. Kaestli, S. König, D. Kotlinski, U. Langenegger, F. Meier, D. Renker, T. Rohe, J. Sibille, L. Bäni, P. Bortignon, M. A. Buchmann, B. Casal, N. Chanon, A. Deisher, G. Dissertori, M. Dittmar, M. Donegà, M. Dünser, J. Eugster, K. Freudenreich, C. Grab, D. Hits, P. Lecomte, W. Lustermann, A. C. Marini, P. Martinez Ruiz del Arbol, N. Mohr, F. Moortgat, C. Nägeli, P. Nef, F. Nessi-Tedaldi, F. Pandolfi, L. Pape, F. Pauss, M. Peruzzi, F. J. Ronga, M. Rossini, L. Sala, A. K. Sanchez, A. Starodumov, B. Stieger, M. Takahashi, L. Tauscher, A. Thea, K. Theofilatos, D. Treille, C. Urscheler, R. Wallny, H. A. Weber, L. Wehrli, C. Amsler, V. Chiochia, S. De Visscher, C. Favaro, M. Ivova Rikova, B. Millan Mejias, P. Otiougova, P. Robmann, H. Snoek, S. Tupputi, M. Verzetti, Y. H. Chang, K. H. Chen, C. M. Kuo, S. W. Li, W. Lin, Z. K. Liu, Y. J. Lu, D. Mekterovic, A. P. Singh, R. Volpe, S. S. Yu, P. Bartalini, P. Chang, Y. H. Chang, Y. W. Chang, Y. Chao, K. F. Chen, C. Dietz, U. Grundler, W.-S. Hou, Y. Hsiung, K. Y. Kao, Y. J. Lei, R.-S. Lu, D. Majumder, E. Petrakou, X. Shi, J. G. Shiu, Y. M. Tzeng, X. Wan, M. Wang, B. Asavapibhop, N. Srimanobhas, A. Adiguzel, M. N. Bakirci, S. Cerci, C. Dozen, I. Dumanoglu, E. Eskut, S. Girgis, G. Gokbulut, E. Gurpinar, I. Hos, E. E. Kangal, T. Karaman, G. Karapinar, A. Kayis Topaksu, G. Onengut, K. Ozdemir, S. Ozturk, A. Polatoz, K. Sogut, D. Sunar Cerci, B. Tali, H. Topakli, L. N. Vergili, M. Vergili, I. V. Akin, T. Aliev, B. Bilin, S. Bilmis, M. Deniz, H. Gamsizkan, A. M. Guler, K. Ocalan, A. Ozpineci, M. Serin, R. Sever, U. E. Surat, M. Yalvac, E. Yildirim, M. Zeyrek, E. Gülmez, B. Isildak, M. Kaya, O. Kaya, S. Ozkorucuklu, N. Sonmez, K. Cankocak, L. Levchuk, F. Bostock, J. J. Brooke, E. Clement, D. Cussans, H. Flacher, R. Frazier, J. Goldstein, M. Grimes, G. P. Heath, H. F. Heath, L. Kreczko, S. Metson, D. M. Newbold, K. Nirunpong, A. Poll, S. Senkin, V. J. Smith, T. Williams, L. Basso, K. W. Bell, A. Belyaev, C. Brew, R. M. Brown, D. J. A. Cockerill, J. A. Coughlan, K. Harder, S. Harper, J. Jackson, B. W. Kennedy, E. Olaiya, D. Petyt, B. C. Radburn-Smith, C. H. Shepherd-Themistocleous, I. R. Tomalin, W. J. Womersley, R. Bainbridge, G. Ball, R. Beuselinck, O. Buchmuller, D. Colling, N. Cripps, M. Cutajar, P. Dauncey, G. Davies, M. Della Negra, W. Ferguson, J. Fulcher, D. Futyan, A. Gilbert, A. Guneratne Bryer, G. Hall, Z. Hatherell, J. Hays, G. Iles, M. Jarvis, G. Karapostoli, L. Lyons, A.-M. Magnan, J. Marrouche, B. Mathias, R. Nandi, J. Nash, A. Nikitenko, A. Papageorgiou, J. Pela, M. Pesaresi, K. Petridis, M. Pioppi, D. M. Raymond, S. Rogerson, A. Rose, M. J. Ryan, C. Seez, P. Sharp, A. Sparrow, M. Stoye, A. Tapper, M. Vazquez Acosta, T. Virdee, S. Wakefield, N. Wardle, T. Whyntie, M. Chadwick, J. E. Cole, P. R. Hobson, A. Khan, P. Kyberd, D. Leggat, D. Leslie, W. Martin, I. D. Reid, P. Symonds, L. Teodorescu, M. Turner, K. Hatakeyama, H. Liu, T. Scarborough, O. Charaf, C. Henderson, P. Rumerio, A. Avetisyan, T. Bose, C. Fantasia, A. Heister, P. Lawson, D. Lazic, J. Rohlf, D. Sperka, J. St. John, L. Sulak, J. Alimena, S. Bhattacharya, D. Cutts, A. Ferapontov, U. Heintz, S. Jabeen, G. Kukartsev, E. Laird, G. Landsberg, M. Luk, M. Narain, D. Nguyen, M. Segala, T. Sinthuprasith, T. Speer, K. V. Tsang, R. Breedon, G. Breto, M. Calderon De La Barca Sanchez, S. Chauhan, M. Chertok, J. Conway, R. Conway, P. T. Cox, J. Dolen, R. Erbacher, M. Gardner, R. Houtz, W. Ko, A. Kopecky, R. Lander, T. Miceli, D. Pellett, F. Ricci-Tam, B. Rutherford, M. Searle, J. Smith, M. Squires, M. Tripathi, R. Vasquez Sierra, V. Andreev, D. Cline, R. Cousins, J. Duris, S. Erhan, P. Everaerts, C. Farrell, J. Hauser, M. Ignatenko, C. Jarvis, C. Plager, G. Rakness, P. Schlein, P. Traczyk, V. Valuev, M. Weber, J. Babb, R. Clare, M. E. Dinardo, J. Ellison, J. W. Gary, F. Giordano, G. Hanson, G. Y. Jeng, H. Liu, O. R. Long, A. Luthra, H. Nguyen, S. Paramesvaran, J. Sturdy, S. Sumowidagdo, R. Wilken, S. Wimpenny, W. Andrews, J. G. Branson, G. B. Cerati, S. Cittolin, D. Evans, F. Golf, A. Holzner, R. Kelley, M. Lebourgeois, J. Letts, I. Macneill, B. Mangano, S. Padhi, C. Palmer, G. Petrucciani, M. Pieri, M. Sani, V. Sharma, S. Simon, E. Sudano, M. Tadel, Y. Tu, A. Vartak, S. Wasserbaech, F. Würthwein, A. Yagil, J. Yoo, D. Barge, R. Bellan, C. Campagnari, M. D’Alfonso, T. Danielson, K. Flowers, P. Geffert, J. Incandela, C. Justus, P. Kalavase, S. A. Koay, D. Kovalskyi, V. Krutelyov, S. Lowette, N. Mccoll, V. Pavlunin, F. Rebassoo, J. Ribnik, J. Richman, R. Rossin, D. Stuart, W. To, C. West, A. Apresyan, A. Bornheim, Y. Chen, E. Di Marco, J. Duarte, M. Gataullin, Y. Ma, A. Mott, H. B. Newman, C. Rogan, M. Spiropulu, V. Timciuc, J. Veverka, R. Wilkinson, S. Xie, Y. Yang, R. Y. Zhu, B. Akgun, V. Azzolini, A. Calamba, R. Carroll, T. Ferguson, Y. Iiyama, D. W. Jang, Y. F. Liu, M. Paulini, H. Vogel, I. Vorobiev, J. P. Cumalat, B. R. Drell, C. J. Edelmaier, W. T. Ford, A. Gaz, B. Heyburn, E. Luiggi Lopez, J. G. Smith, K. Stenson, K. A. Ulmer, S. R. Wagner, J. Alexander, A. Chatterjee, N. Eggert, L. K. Gibbons, B. Heltsley, A. Khukhunaishvili, B. Kreis, N. Mirman, G. Nicolas Kaufman, J. R. Patterson, A. Ryd, E. Salvati, W. Sun, W. D. Teo, J. Thom, J. Thompson, J. Tucker, J. Vaughan, Y. Weng, L. Winstrom, P. Wittich, D. Winn, S. Abdullin, M. Albrow, J. Anderson, L. A. T. Bauerdick, A. Beretvas, J. Berryhill, P. C. Bhat, I. Bloch, K. Burkett, J. N. Butler, V. Chetluru, H. W. K. Cheung, F. Chlebana, V. D. Elvira, I. Fisk, J. Freeman, Y. Gao, D. Green, O. Gutsche, J. Hanlon, R. M. Harris, J. Hirschauer, B. Hooberman, S. Jindariani, M. Johnson, U. Joshi, B. Kilminster, B. Klima, S. Kunori, S. Kwan, C. Leonidopoulos, J. Linacre, D. Lincoln, R. Lipton, J. Lykken, K. Maeshima, J. M. Marraffino, S. Maruyama, D. Mason, P. McBride, K. Mishra, S. Mrenna, Y. Musienko, C. Newman-Holmes, V. O’Dell, O. Prokofyev, E. Sexton-Kennedy, S. Sharma, W. J. Spalding, L. Spiegel, P. Tan, L. Taylor, S. Tkaczyk, N. V. Tran, L. Uplegger, E. W. Vaandering, R. Vidal, J. Whitmore, W. Wu, F. Yang, F. Yumiceva, J. C. Yun, D. Acosta, P. Avery, D. Bourilkov, M. Chen, T. Cheng, S. Das, M. De Gruttola, G. P. Di Giovanni, D. Dobur, A. Drozdetskiy, R. D. Field, M. Fisher, Y. Fu, I. K. Furic, J. Gartner, J. Hugon, B. Kim, J. Konigsberg, A. Korytov, A. Kropivnitskaya, T. Kypreos, J. F. Low, K. Matchev, P. Milenovic, G. Mitselmakher, L. Muniz, M. Park, R. Remington, A. Rinkevicius, P. Sellers, N. Skhirtladze, M. Snowball, J. Yelton, M. Zakaria, V. Gaultney, S. Hewamanage, L. M. Lebolo, S. Linn, P. Markowitz, G. Martinez, J. L. Rodriguez, T. Adams, A. Askew, J. Bochenek, J. Chen, B. Diamond, S. V. Gleyzer, J. Haas, S. Hagopian, V. Hagopian, M. Jenkins, K. F. Johnson, H. Prosper, V. Veeraraghavan, M. Weinberg, M. M. Baarmand, B. Dorney, M. Hohlmann, H. Kalakhety, I. Vodopiyanov, M. R. Adams, I. M. Anghel, L. Apanasevich, Y. Bai, V. E. Bazterra, R. R. Betts, I. Bucinskaite, J. Callner, R. Cavanaugh, O. Evdokimov, L. Gauthier, C. E. Gerber, D. J. Hofman, S. Khalatyan, F. Lacroix, M. Malek, C. O’Brien, C. Silkworth, D. Strom, P. Turner, N. Varelas, U. Akgun, E. A. Albayrak, B. Bilki, W. Clarida, F. Duru, S. Griffiths, J.-P. Merlo, H. Mermerkaya, A. Mestvirishvili, A. Moeller, J. Nachtman, C. R. Newsom, E. Norbeck, Y. Onel, F. Ozok, S. Sen, E. Tiras, J. Wetzel, T. Yetkin, K. Yi, B. A. Barnett, B. Blumenfeld, S. Bolognesi, D. Fehling, G. Giurgiu, A. V. Gritsan, Z. J. Guo, G. Hu, P. Maksimovic, S. Rappoccio, M. Swartz, A. Whitbeck, P. Baringer, A. Bean, G. Benelli, R. P. Kenny Iii, M. Murray, D. Noonan, S. Sanders, R. Stringer, G. Tinti, J. S. Wood, V. Zhukova, A. F. Barfuss, T. Bolton, I. Chakaberia, A. Ivanov, S. Khalil, M. Makouski, Y. Maravin, S. Shrestha, I. Svintradze, J. Gronberg, D. Lange, D. Wright, A. Baden, M. Boutemeur, B. Calvert, S. C. Eno, J. A. Gomez, N. J. Hadley, R. G. Kellogg, M. Kirn, T. Kolberg, Y. Lu, M. Marionneau, A. C. Mignerey, K. Pedro, A. Peterman, A. Skuja, J. Temple, M. B. Tonjes, S. C. Tonwar, E. Twedt, A. Apyan, G. Bauer, J. Bendavid, W. Busza, E. Butz, I. A. Cali, M. Chan, V. Dutta, G. Gomez Ceballos, M. Goncharov, K. A. Hahn, Y. Kim, M. Klute, K. Krajczar, W. Li, P. D. Luckey, T. Ma, S. Nahn, C. Paus, D. Ralph, C. Roland, G. Roland, M. Rudolph, G. S. F. Stephans, F. Stöckli, K. Sumorok, K. Sung, D. Velicanu, E. A. Wenger, R. Wolf, B. Wyslouch, M. Yang, Y. Yilmaz, A. S. Yoon, M. Zanetti, S. I. Cooper, B. Dahmes, A. De Benedetti, G. Franzoni, A. Gude, S. C. Kao, K. Klapoetke, Y. Kubota, J. Mans, N. Pastika, R. Rusack, M. Sasseville, A. Singovsky, N. Tambe, J. Turkewitz, L. M. Cremaldi, R. Kroeger, L. Perera, R. Rahmat, D. A. Sanders, E. Avdeeva, K. Bloom, S. Bose, J. Butt, D. R. Claes, A. Dominguez, M. Eads, J. Keller, I. Kravchenko, J. Lazo-Flores, H. Malbouisson, S. Malik, G. R. Snow, U. Baur, A. Godshalk, I. Iashvili, S. Jain, A. Kharchilava, A. Kumar, S. P. Shipkowski, K. Smith, G. Alverson, E. Barberis, D. Baumgartel, M. Chasco, J. Haley, D. Nash, D. Trocino, D. Wood, J. Zhang, A. Anastassov, A. Kubik, N. Mucia, N. Odell, R. A. Ofierzynski, B. Pollack, A. Pozdnyakov, M. Schmitt, S. Stoynev, M. Velasco, S. Won, L. Antonelli, D. Berry, A. Brinkerhoff, M. Hildreth, C. Jessop, D. J. Karmgard, J. Kolb, K. Lannon, W. Luo, S. Lynch, N. Marinelli, D. M. Morse, T. Pearson, M. Planer, R. Ruchti, J. Slaunwhite, N. Valls, M. Wayne, M. Wolf, B. Bylsma, L. S. Durkin, C. Hill, R. Hughes, K. Kotov, T. Y. Ling, D. Puigh, M. Rodenburg, C. Vuosalo, G. Williams, B. L. Winer, N. Adam, E. Berry, P. Elmer, D. Gerbaudo, V. Halyo, P. Hebda, J. Hegeman, A. Hunt, P. Jindal, D. Lopes Pegna, P. Lujan, D. Marlow, T. Medvedeva, M. Mooney, J. Olsen, P. Piroué, X. Quan, A. Raval, B. Safdi, H. Saka, D. Stickland, C. Tully, J. S. Werner, A. Zuranski, J. G. Acosta, E. Brownson, X. T. Huang, A. Lopez, H. Mendez, S. Oliveros, J. E. Ramirez Vargas, A. Zatserklyaniy, E. Alagoz, V. E. Barnes, D. Benedetti, G. Bolla, D. Bortoletto, M. De Mattia, A. Everett, Z. Hu, M. Jones, O. Koybasi, M. Kress, A. T. Laasanen, N. Leonardo, V. Maroussov, P. Merkel, D. H. Miller, N. Neumeister, I. Shipsey, D. Silvers, A. Svyatkovskiy, M. Vidal Marono, H. D. Yoo, J. Zablocki, Y. Zheng, S. Guragain, N. Parashar, A. Adair, C. Boulahouache, K. M. Ecklund, F. J. M. Geurts, B. P. Padley, R. Redjimi, J. Roberts, J. Zabel, B. Betchart, A. Bodek, Y. S. Chung, R. Covarelli, P. de Barbaro, R. Demina, Y. Eshaq, T. Ferbel, A. Garcia-Bellido, P. Goldenzweig, J. Han, A. Harel, D. C. Miner, D. Vishnevskiy, M. Zielinski, A. Bhatti, R. Ciesielski, L. Demortier, K. Goulianos, G. Lungu, S. Malik, C. Mesropian, S. Arora, A. Barker, J. P. Chou, C. Contreras-Campana, E. Contreras-Campana, D. Duggan, D. Ferencek, Y. Gershtein, R. Gray, E. Halkiadakis, D. Hidas, A. Lath, S. Panwalkar, M. Park, R. Patel, V. Rekovic, J. Robles, K. Rose, S. Salur, S. Schnetzer, C. Seitz, S. Somalwar, R. Stone, S. Thomas, G. Cerizza, M. Hollingsworth, S. Spanier, Z. C. Yang, A. York, R. Eusebi, W. Flanagan, J. Gilmore, T. Kamon, V. Khotilovich, R. Montalvo, I. Osipenkov, Y. Pakhotin, A. Perloff, J. Roe, A. Safonov, T. Sakuma, S. Sengupta, I. Suarez, A. Tatarinov, D. Toback, N. Akchurin, J. Damgov, C. Dragoiu, P. R. Dudero, C. Jeong, K. Kovitanggoon, S. W. Lee, T. Libeiro, Y. Roh, I. Volobouev, E. Appelt, A. G. Delannoy, C. Florez, S. Greene, A. Gurrola, W. Johns, C. Johnston, P. Kurt, C. Maguire, A. Melo, M. Sharma, P. Sheldon, B. Snook, S. Tuo, J. Velkovska, M. W. Arenton, M. Balazs, S. Boutle, B. Cox, B. Francis, J. Goodell, R. Hirosky, A. Ledovskoy, C. Lin, C. Neu, J. Wood, R. Yohay, S. Gollapinni, R. Harr, P. E. Karchin, C. Kottachchi Kankanamge Don, P. Lamichhane, A. Sakharov, M. Anderson, D. A. Belknap, L. Borrello, D. Carlsmith, M. Cepeda, S. Dasu, E. Friis, L. Gray, K. S. Grogg, M. Grothe, R. Hall-Wilton, M. Herndon, A. Hervé, P. Klabbers, J. Klukas, A. Lanaro, C. Lazaridis, J. Leonard, R. Loveless, A. Mohapatra, I. Ojalvo, F. Palmonari, G. A. Pierro, I. Ross, A. Savin, W. H. Smith, J. Swanson

**Affiliations:** 1 CERN, Geneva, Switzerland; 2 Yerevan Physics Institute, Yerevan, Armenia; 3 Institut für Hochenergiephysik der OeAW, Wien, Austria; 4 National Centre for Particle and High Energy Physics, Minsk, Belarus; 5 Universiteit Antwerpen, Antwerpen, Belgium; 6 Vrije Universiteit Brussel, Brussel, Belgium; 7 Université Libre de Bruxelles, Bruxelles, Belgium; 8 Ghent University, Ghent, Belgium; 9 Université Catholique de Louvain, Louvain-la-Neuve, Belgium; 10 Université de Mons, Mons, Belgium; 11 Centro Brasileiro de Pesquisas Fisicas, Rio de Janeiro, Brazil; 12 Universidade do Estado do Rio de Janeiro, Rio de Janeiro, Brazil; 13 Universidade Estadual Paulista, São Paulo, Brazil; 14 Universidade Federal do ABC, São Paulo, Brazil; 15 Institute for Nuclear Research and Nuclear Energy, Sofia, Bulgaria; 16 University of Sofia, Sofia, Bulgaria; 17 Institute of High Energy Physics, Beijing, China; 18 State Key Laboratory of Nuclear Physics and Technology, Peking University, Beijing, China; 19 Universidad de Los Andes, Bogota, Colombia; 20 Technical University of Split, Split, Croatia; 21 University of Split, Split, Croatia; 22 Institute Rudjer Boskovic, Zagreb, Croatia; 23 University of Cyprus, Nicosia, Cyprus; 24 Charles University, Prague, Czech Republic; 25 Academy of Scientific Research and Technology of the Arab Republic of Egypt, Egyptian Network of High Energy Physics, Cairo, Egypt; 26 National Institute of Chemical Physics and Biophysics, Tallinn, Estonia; 27 Department of Physics, University of Helsinki, Helsinki, Finland; 28 Helsinki Institute of Physics, Helsinki, Finland; 29 Lappeenranta University of Technology, Lappeenranta, Finland; 30 DSM/IRFU, CEA/Saclay, Gif-sur-Yvette, France; 31 Laboratoire Leprince-Ringuet, Ecole Polytechnique, IN2P3-CNRS, Palaiseau, France; 32 Institut Pluridisciplinaire Hubert Curien, Université de Strasbourg, Université de Haute Alsace Mulhouse, CNRS/IN2P3, Strasbourg, France; 33 Centre de Calcul de l’Institut National de Physique Nucleaire et de Physique des Particules, CNRS/IN2P3, Villeurbanne, France; 34 Université de Lyon, Université Claude Bernard Lyon 1, Institut de Physique Nucléaire de Lyon, CNRS-IN2P3, Villeurbanne, France; 35 Institute of High Energy Physics and Informatization, Tbilisi State University, Tbilisi, Georgia; 36 I. Physikalisches Institut, RWTH Aachen University, Aachen, Germany; 37 III. Physikalisches Institut A, RWTH Aachen University, Aachen, Germany; 38 III. Physikalisches Institut B, RWTH Aachen University, Aachen, Germany; 39 Deutsches Elektronen-Synchrotron, Hamburg, Germany; 40 University of Hamburg, Hamburg, Germany; 41 Institut für Experimentelle Kernphysik, Karlsruhe, Germany; 42 Institute of Nuclear Physics “Demokritos”, Aghia Paraskevi, Greece; 43 University of Athens, Athens, Greece; 44 University of Ioánnina, Ioánnina, Greece; 45 KFKI Research Institute for Particle and Nuclear Physics, Budapest, Hungary; 46 Institute of Nuclear Research ATOMKI, Debrecen, Hungary; 47 University of Debrecen, Debrecen, Hungary; 48 Panjab University, Chandigarh, India; 49 University of Delhi, Delhi, India; 50 Saha Institute of Nuclear Physics, Kolkata, India; 51 Bhabha Atomic Research Centre, Mumbai, India; 52 Tata Institute of Fundamental Research - EHEP, Mumbai, India; 53 Tata Institute of Fundamental Research - HECR, Mumbai, India; 54 Institute for Research in Fundamental Sciences (IPM), Tehran, Iran; 55 INFN Sezione di Bari, Bari, Italy; 56 Università di Bari, Bari, Italy; 57 Politecnico di Bari, Bari, Italy; 58 INFN Sezione di Bologna, Bologna, Italy; 59 Università di Bologna, Bologna, Italy; 60 INFN Sezione di Catania, Catania, Italy; 61 Università di Catania, Catania, Italy; 62 INFN Sezione di Firenze, Firenze, Italy; 63 Università di Firenze, Firenze, Italy; 64 INFN Laboratori Nazionali di Frascati, Frascati, Italy; 65 INFN Sezione di Genova, Genova, Italy; 66 Università di Genova, Genova, Italy; 67 INFN Sezione di Milano-Bicocca, Milano, Italy; 68 Università di Milano-Bicocca, Milano, Italy; 69 INFN Sezione di Napoli, Napoli, Italy; 70 Università di Napoli ’Federico II’, Napoli, Italy; 71 Università della Basilicata (Potenza), Napoli, Italy; 72 Università G. Marconi (Roma), Napoli, Italy; 73 INFN Sezione di Padova, Padova, Italy; 74 Università di Padova, Padova, Italy; 75 Università di Trento (Trento), Padova, Italy; 76 INFN Sezione di Pavia, Pavia, Italy; 77 Università di Pavia, Pavia, Italy; 78 INFN Sezione di Perugia, Perugia, Italy; 79 Università di Perugia, Perugia, Italy; 80 INFN Sezione di Pisa, Pisa, Italy; 81 Università di Pisa, Pisa, Italy; 82 Scuola Normale Superiore di Pisa, Pisa, Italy; 83 INFN Sezione di Roma, Roma, Italy; 84 Università di Roma, Roma, Italy; 85 INFN Sezione di Torino, Torino, Italy; 86 Università di Torino, Torino, Italy; 87 Università del Piemonte Orientale (Novara), Torino, Italy; 88 INFN Sezione di Trieste, Trieste, Italy; 89 Università di Trieste, Trieste, Italy; 90 Kangwon National University, Chunchon, Korea; 91 Kyungpook National University, Daegu, Korea; 92 Institute for Universe and Elementary Particles, Chonnam National University, Kwangju, Korea; 93 Korea University, Seoul, Korea; 94 University of Seoul, Seoul, Korea; 95 Sungkyunkwan University, Suwon, Korea; 96 Vilnius University, Vilnius, Lithuania; 97 Centro de Investigacion y de Estudios Avanzados del IPN, Mexico City, Mexico; 98 Universidad Iberoamericana, Mexico City, Mexico; 99 Benemerita Universidad Autonoma de Puebla, Puebla, Mexico; 100 Universidad Autónoma de San Luis Potosí, San Luis Potosí, Mexico; 101 University of Auckland, Auckland, New Zealand; 102 University of Canterbury, Christchurch, New Zealand; 103 National Centre for Physics, Quaid-I-Azam University, Islamabad, Pakistan; 104 National Centre for Nuclear Research, Swierk, Poland; 105 Institute of Experimental Physics, Faculty of Physics, University of Warsaw, Warsaw, Poland; 106 Laboratório de Instrumentação e Física Experimental de Partículas, Lisboa, Portugal; 107 Joint Institute for Nuclear Research, Dubna, Russia; 108 Petersburg Nuclear Physics Institute, Gatchina (St. Petersburg), Russia; 109 Institute for Nuclear Research, Moscow, Russia; 110 Institute for Theoretical and Experimental Physics, Moscow, Russia; 111 P.N. Lebedev Physical Institute, Moscow, Russia; 112 Skobeltsyn Institute of Nuclear Physics, Lomonosov Moscow State University, Moscow, Russia; 113 State Research Center of Russian Federation, Institute for High Energy Physics, Protvino, Russia; 114 Faculty of Physics and Vinca Institute of Nuclear Sciences, University of Belgrade, Belgrade, Serbia; 115 Centro de Investigaciones Energéticas Medioambientales y Tecnológicas (CIEMAT), Madrid, Spain; 116 Universidad Autónoma de Madrid, Madrid, Spain; 117 Universidad de Oviedo, Oviedo, Spain; 118 Instituto de Física de Cantabria (IFCA), CSIC-Universidad de Cantabria, Santander, Spain; 119 CERN, European Organization for Nuclear Research, Geneva, Switzerland; 120 Paul Scherrer Institut, Villigen, Switzerland; 121 Institute for Particle Physics, ETH Zurich, Zurich, Switzerland; 122 Universität Zürich, Zurich, Switzerland; 123 National Central University, Chung-Li, Taiwan; 124 National Taiwan University (NTU), Taipei, Taiwan; 125 Chulalongkorn University, Bangkok, Thailand; 126 Cukurova University, Adana, Turkey; 127 Physics Department, Middle East Technical University, Ankara, Turkey; 128 Bogazici University, Istanbul, Turkey; 129 Istanbul Technical University, Istanbul, Turkey; 130 National Scientific Center, Kharkov Institute of Physics and Technology, Kharkov, Ukraine; 131 University of Bristol, Bristol, United Kingdom; 132 Rutherford Appleton Laboratory, Didcot, United Kingdom; 133 Imperial College, London, United Kingdom; 134 Brunel University, Uxbridge, United Kingdom; 135 Baylor University, Waco, USA; 136 The University of Alabama, Tuscaloosa, USA; 137 Boston University, Boston, USA; 138 Brown University, Providence, USA; 139 University of California, Davis, Davis, USA; 140 University of California, Los Angeles, USA; 141 University of California, Riverside, Riverside, USA; 142 University of California, San Diego, La Jolla, USA; 143 University of California, Santa Barbara, Santa Barbara, USA; 144 California Institute of Technology, Pasadena, USA; 145 Carnegie Mellon University, Pittsburgh, USA; 146 University of Colorado at Boulder, Boulder, USA; 147 Cornell University, Ithaca, USA; 148 Fairfield University, Fairfield, USA; 149 Fermi National Accelerator Laboratory, Batavia, USA; 150 University of Florida, Gainesville, USA; 151 Florida International University, Miami, USA; 152 Florida State University, Tallahassee, USA; 153 Florida Institute of Technology, Melbourne, USA; 154 University of Illinois at Chicago (UIC), Chicago, USA; 155 The University of Iowa, Iowa City, USA; 156 Johns Hopkins University, Baltimore, USA; 157 The University of Kansas, Lawrence, USA; 158 Kansas State University, Manhattan, USA; 159 Lawrence Livermore National Laboratory, Livermore, USA; 160 University of Maryland, College Park, USA; 161 Massachusetts Institute of Technology, Cambridge, USA; 162 University of Minnesota, Minneapolis, USA; 163 University of Mississippi, Oxford, USA; 164 University of Nebraska-Lincoln, Lincoln, USA; 165 State University of New York at Buffalo, Buffalo, USA; 166 Northeastern University, Boston, USA; 167 Northwestern University, Evanston, USA; 168 University of Notre Dame, Notre Dame, USA; 169 The Ohio State University, Columbus, USA; 170 Princeton University, Princeton, USA; 171 University of Puerto Rico, Mayaguez, USA; 172 Purdue University, West Lafayette, USA; 173 Purdue University Calumet, Hammond, USA; 174 Rice University, Houston, USA; 175 University of Rochester, Rochester, USA; 176 The Rockefeller University, New York, USA; 177 Rutgers, the State University of New Jersey, Piscataway, USA; 178 University of Tennessee, Knoxville, USA; 179 Texas A&M University, College Station, USA; 180 Texas Tech University, Lubbock, USA; 181 Vanderbilt University, Nashville, USA; 182 University of Virginia, Charlottesville, USA; 183 Wayne State University, Detroit, USA; 184 University of Wisconsin, Madison, USA

## Abstract

A search for physics beyond the standard model is performed with events having one or more hadronically decaying *τ* leptons, highly energetic jets, and large transverse momentum imbalance. The data sample corresponds to an integrated luminosity of 4.98 fb^−1^ of proton-proton collisions at $\sqrt{s}=7 ~\text {TeV} $ collected with the CMS detector at the LHC in 2011. The number of observed events is consistent with predictions for standard model processes. Lower limits on the mass of the gluino in supersymmetric models are determined.

## Introduction

The standard model (SM) of particle physics has been successful in explaining a wide variety of data. In spite of this, the SM is incomplete. For example, it possesses a divergence in the Higgs sector [1] and has no cold dark matter (DM) candidate [2]. Many models of physics beyond the SM (BSM) have been proposed in order to address these problems.

DM particles, if produced in proton-proton collisions at the CERN Large Hadron Collider (LHC), would escape detection and result in a significant transverse momentum (*p*_T_) imbalance in the detector. Additionally, cascade decays of heavy colored particles to final states with a high multiplicity of energetic jets and *τ* leptons appear very naturally in many BSM physics scenarios. Hence, events with multiple *τ* lepton candidates, large jet multiplicity, and significant transverse momentum imbalance, represent a distinct signature of new physics. In this paper, focus is placed on final states with hadronically decaying *τ* leptons. In what follows, the visible part of a hadronically decaying *τ* lepton will be referred to as *τ*_h_.

In certain models of supersymmetry (SUSY), the lightest supersymmetric particle (LSP) is a candidate for DM. It has been appreciated for some time that the DM relic density may be sensitive to coannihilation processes involving the LSP and the next-to-lightest supersymmetric particle (NLSP). Coannihilation is characterized by a mass difference (Δ*M*) between the NLSP and the LSP of approximately 5–15 GeV [3–6]. This small mass difference would be necessary to allow the NLSP to coannihilate with the LSP in the early universe, leading to the dark matter abundance that is currently observed [7]. If the supersymmetric partner of the *τ* lepton, the stau ($\widetilde{ \tau } $), is the NLSP, and if the $\widetilde{ \tau } $ decays primarily to a *τ* lepton and the LSP, small values of Δ*M* would lead to final states with low-energy *τ* leptons (*p*_T_∼Δ*M*) [8]. Decays of colored SUSY particles can produce the $\widetilde{ \tau } $ via chargino ($\widetilde{\chi} ^{\pm}$) or neutralino ($\widetilde{\chi}^{0} $) intermediate states (e.g., $\widetilde{\chi}^{0}_{2} \to\tau \widetilde{ \tau } \to \tau\tau \widetilde{\chi}^{0}_{1} $), resulting in final states with at least one *τ*_h_.

We present a search for BSM particles in events with exactly one *τ*_h_ lepton and jets (single-*τ*_h_ final state), and in events with jets and two or more *τ*_h_ leptons (multiple-*τ*_h_ final state). These two topologies provide complementary sensitivity to models with a wide range of Δ*M* values. For example, in the case of very small values of Δ*M* (∼5 GeV), the low-energy *τ*_h_ cannot be effectively detected and the search for new physics in the single-*τ*_h_ final state has better sensitivity. The analysis is performed using proton-proton collision data at $\sqrt {s} = 7 ~\text{TeV} $ collected with the Compact Muon Solenoid (CMS) detector [9] at the LHC in 2011. The data sample corresponds to an integrated luminosity of 4.98±0.11 fb^−1^. The search is characterized by methods that determine the backgrounds directly from data, to reduce the reliance on simulation. To illustrate the sensitivity of this search to BSM processes, the constrained minimal supersymmetric extension of the standard model, or minimal supergravity, is chosen as the benchmark [3, 10, 11]; we denote this benchmark as “CMSSM”. An interpretation of the results in the context of simplified model spectra (SMS) [12, 13] is also presented. The ATLAS collaboration has published a result on a search for one or more hadronically decaying tau leptons, highly energetic jets, and a large transverse momentum imbalance probing minimal Gauge Mediated Symmetry Breaking (GMSB) models [14].

## The CMS detector

The central feature of the CMS apparatus is a superconducting solenoid, of 6 m inner diameter, providing a magnetic field of 3.8 T. Within the field volume are a silicon pixel and strip tracker, a crystal electromagnetic calorimeter (ECAL), which includes a silicon sensor preshower detector in front of the ECAL endcaps, and a brass-scintillator hadron calorimeter. Muons are measured in gas-ionization detectors embedded in the steel return yoke. In addition to the barrel and endcap detectors, CMS has extensive forward calorimetry.

The inner tracker measures charged particles within |*η*|<2.5 and provides an impact parameter resolution of about 15 μm and a *p*_T_ resolution of about 1.5 % for 100 GeV particles. Collision events are selected with a first-level trigger based on fast electronics, and a higher-level trigger that runs a version of the offline reconstruction program optimized for speed.

The CMS experiment uses a right-handed coordinate system, with the origin at the nominal interaction point, the *x* axis pointing to the center of the LHC ring, the *y* axis pointing up (perpendicular to the plane of the LHC ring), and the *z* axis along the counterclockwise beam direction. The polar angle *θ* is measured from the positive *z* axis and the azimuthal angle in the *x*–*y* plane. The pseudorapidity is given by *η*=−ln[tan(*θ*/2)].

## Object reconstruction and identification

Jets in the detector are reconstructed using particle-flow (PF) objects [15]. In the PF approach, information from all subdetectors is combined to reconstruct and identify final-state particles (muons, electrons, photons, and charged and neutral hadrons) produced in the collision. The anti-*k*_T_ clustering algorithm [16] with a distance parameter *R*=0.5 is used for jet clustering. Jets are required to satisfy criteria designed to identify anomalous behavior in the calorimeters, and to be well separated from any identified *τ* lepton.

Validation and efficiency studies are performed utilizing events with a *τ*_h_ lepton and a light-lepton *ℓ*, with *ℓ* representing an electron (e) or muon (*μ*). Muons are reconstructed using the tracker and muon chambers. Selection requirements based on the minimum number of hits in the silicon tracker, pixel detector, and muon chambers are applied to suppress muon backgrounds from decays-in-flight of pions or kaons [17]. Electrons are reconstructed by combining tracks with ECAL clusters. Requirements are imposed to distinguish between prompt and non-prompt electrons, where the latter can arise from charged pion decay or photon conversion [18]. The light-lepton candidates are required to satisfy both track and ECAL isolation requirements. The track isolation variable is defined as the sum of the *p*_T_ of the tracks, as measured by the tracking system, within an isolation cone of radius $\Delta R = \sqrt{(\Delta\eta)^{2} + (\Delta\phi)^{2}}=0.4$ centered on the light-lepton track. The ECAL isolation variable is based on the amount of energy deposited in the ECAL within the same isolation cone. In both cases the contribution from the light-lepton candidate is removed from the sum.

Reconstruction of hadronically decaying *τ* leptons is performed using the hadron-plus-strips (HPS) algorithm [19], designed to optimize the performance of *τ*_h_ reconstruction by considering specific *τ*_h_ decay modes. To suppress backgrounds in which light-quark or gluon jets mimic hadronic *τ* decays, a *τ*_h_ candidate is required to be spatially isolated from other energy deposits in the calorimeter. Charged hadrons and photons not considered in the reconstruction of the *τ*_h_ decay mode are used to calculate the isolation. Additionally, *τ*_h_ candidates are required to be distinguished from electrons and muons in the event. In this analysis, two HPS isolation definitions are used. The *τ*_h_ isolation definition used for single-*τ*_h_ final states rejects a *τ*_h_ candidate if one or more charged hadrons with *p*_T_>1.0 GeV or one or more photons with transverse energy *E*_T_>1.5 GeV is found within an isolation cone of radius Δ*R*=0.5. The *τ*_h_ isolation definition used for multiple-*τ*_h_ final states rejects a *τ*_h_ candidate if one or more charged hadrons with *p*_T_>1.5 GeV or one or more photons with transverse energy *E*_T_>2.0 GeV is found within an isolation cone of radius Δ*R*=0.3. The isolation criteria used for the multiple-*τ*_h_ final state increases the signal-to-background ratio while reducing the rate of *τ*_h_ misidentification. This affects the yield of events with light-quark or gluon jets that are misidentified as *τ*_h_ leptons, which depends on the square of the misidentification rate. Here a final state with exactly two *τ*_h_ candidates is considered since events with more than two *τ*_h_ candidates are only a small fraction (<1 %) of events.

The missing transverse momentum  is defined as: 1 where the sum runs over all the jets with $p_{\mathrm {T}} ^{\text{jet}} > 30 ~\text{GeV} $ inside the fiducial detector volume of |*η*|<5. The vector  is the negative of the vector sum in Eq. (1). The observable $H_{\mathrm{T}} = \sum {\boldsymbol{p}}_{\mathrm{T}} ^{\text{jet}}$ is used to estimate the overall energy scale of the event. For the single-*τ*_h_ final state, *H*_T_ is calculated using jets with *p*_T_>50 GeV and will be referred to as $H_{\mathrm {T}} ^{50}$. For the multiple-*τ*_h_ final state, *H*_T_ is calculated using jets with *p*_T_>30 GeV and will be referred to as $H_{\mathrm {T}} ^{30}$. In both instances of the *H*_T_ calculation, we consider all jets in |*η*|<5 (the fiducial detector limit). The use of a lower *p*_T_ threshold for the jets in the multiple-*τ*_h_ final state increases the efficiency of signal events without significantly increasing the background.

## Signal and background samples

The major sources of SM background are top-quark pair ($\mathrm {t}\overline{\mathrm {t}}$) events and events with a W or Z boson accompanied by jets. Both $\mathrm {t}\overline{\mathrm {t}}$ and W+jets events can have genuine *τ*_h_ leptons, large genuine  from W boson decays, and jets that can be misidentified as a *τ*_h_. Similarly, Z+jets events with Z(→*νν*) and with one or more jets misidentified as a *τ*_h_ lepton provide a source of background. Z+jets events with Z(→*νν*) present a background because of the genuine *τ*_h_ leptons and the genuine  from the neutrinos in the *τ*_h_ decay. QCD multijet events can become a background when a mismeasured jet gives rise to large  and jets are misidentified as *τ*_h_ leptons.

Data are compared with predictions obtained from samples of Monte Carlo (MC) simulated events. Signal and background MC samples are produced with the pythia 6.4.22 [20] and MadGraph [21] generators using the Z2 tune [22] and the NLO CTEQ6L1 parton distribution function (PDF) set [23]. The *τ* lepton decays are simulated with the Tauola [24] program. The generated events are processed with a detailed simulation of the CMS apparatus using the Geant4 package [25]. The MC yields are normalized to the integrated luminosity of the data using next-to-leading order (NLO) cross sections [26–31]. For the 2011 LHC running conditions, the mean number of interactions in a single beam crossing is ∼10. The effect of multiple interactions per bunch crossing (pileup) is taken into account by superimposing MC minimum-bias events so that the probability distribution for overlapping pp collisions in the simulation matches the measured distribution.

## Event selection

Events for both the single- and multiple-*τ*_h_ final states are selected using a trigger that requires . This trigger allows us to maintain sensitivity in regions where the *p*_T_ value of the *τ*_h_ is small (*p*_T_∼15 GeV). This trigger efficiency, for an offline selection requirement of , is 98.9 %. For the *τ*_h_ efficiency and validation studies, samples are chosen using triggers that require the presence of both a *τ*_h_ candidate and a muon.

The *τ*_h_ candidates must satisfy *p*_T_>15 GeV and |*η*|<2.1. For the single-*τ*_h_ final state we require that no additional light leptons be present in the event. This requirement suppresses background from $\mathrm {t}\overline{\mathrm {t}} $, W+jets, and Z+jets events. For the multiple-*τ*_h_ final state there is no requirement placed on the number of light leptons.

For the single-*τ*_h_ final state, we define a baseline event selection $H_{\mathrm{T}} ^{50} > 350 ~\text{GeV} $ and . The sample obtained with the baseline selection is used to validate the background predictions. The signal region (SR) for the single-*τ*_h_ final state is defined by $H_{\mathrm{T}} ^{50} > 600 ~\text {GeV} $ and .

For the multiple-*τ*_h_ final state, the SR is defined by  and by the requirement that there be at least two jets with *p*_T_>100 GeV and |*η*|<3.0. QCD multijet events are rejected by requiring the azimuthal difference  between the second leading jet in *p*_T_ and  to satisfy  Finally, events are required to contain at least one *τ*_h_ *τ*_h_ pair separated by Δ*R*(*τ*_h,*i*_,*τ*_h,*j*_)>0.3.

## Background estimate

The background contributions are categorized differently for the single- and multiple-*τ*_h_ final states. For the single-*τ*_h_ final state, the background contributions are divided into events containing a genuine *τ*_h_ and events where a jet is misidentified as a *τ*_h_. For the multiple-*τ*_h_ final state, the main background contribution arises from misidentified *τ*_h_ leptons. We identify the different sources of background individually using dedicated data control regions (CR).

### Estimate of backgrounds in the single-*τ*_h_ final state

In the single-*τ*_h_ final state, the largest background contribution comes from W + jets events that contain a genuine *τ*_h_ lepton. The other significant contribution arises from QCD multijet events in which a jet is misidentified as a *τ*_h_. The W+jets background contribution is estimated using a sample of W+jets events with W→*μν*. The QCD multijet background is determined by selecting a QCD-dominated CR and evaluating the *τ*_h_ misidentification rate.

#### Estimate of the W + jets background in the single-*τ*_h_ final state

To evaluate the W+jets background, we exploit the similarity between W decays to a muon and to a tau lepton and select a sample of W+jets events with W(→*μν*). This sample will be referred to as the muon control sample. To select the muon control sample, events are required to contain exactly one muon and no reconstructed *τ*_h_ or electron. To emulate the *τ*_h_ acceptance, the muon is required to satisfy |*η*|<2.1. The yields in the muon control sample are corrected for muon reconstruction ($\varepsilon_{\mu}^{\text{reco}}$) and isolation efficiency ($\varepsilon_{\mu}^{\text{iso}}$). The muon reconstruction efficiency is derived from data using a sample of Z + jets events and parameterized as a function of *p*_T_ and *η*. The muon isolation criteria help to distinguish between muons from the decay of the W boson and muons from semileptonic decays of c and b quarks. The isolation efficiency is parameterized as a function of the separation from the nearest jet and the momentum of the jet. A correction factor ($P^{ \mathrm{W} }_{\mu}$) is applied to the muons in the muon control sample to account for muons that do not come from a *τ*-lepton decay. This correction factor depends on the *p*_T_ of the muon and the  value in the event and is derived from a simulated sample of W+jets events.

**Fig. 1:**
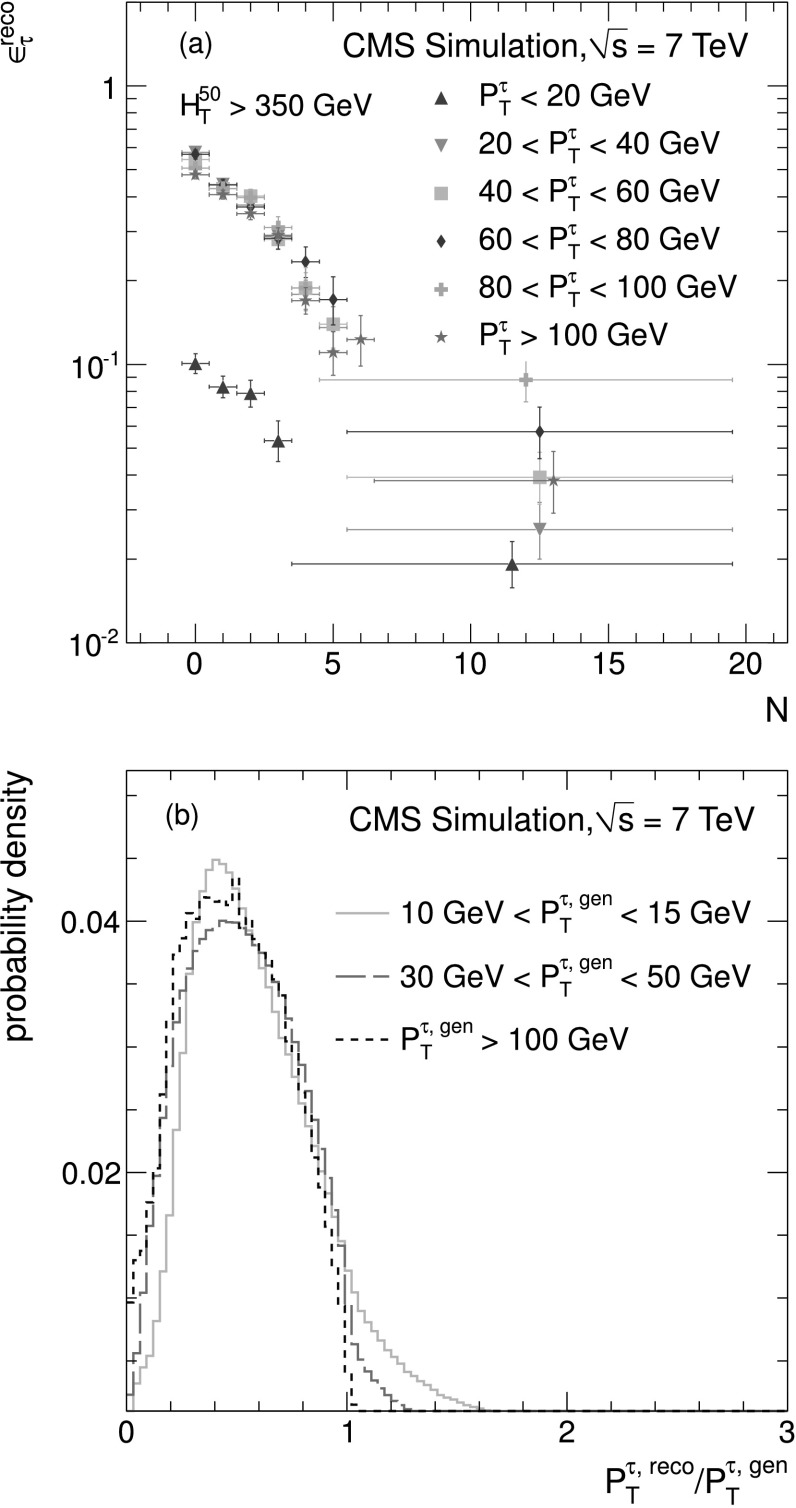
(**a**) Dependence of the *τ*_h_ reconstruction efficiency $\epsilon_{\tau}^{\text{reco}}$ on the number of additional particles *N* in the isolation cone in bins of *τ*_h_ lepton *p*_T_ for the single-*τ*_h_ final state, where *N* is the total number of the photons and charged hadrons in the isolation cone, and (**b**) dependence of *τ*_h_ response on $p_{\mathrm{T}} ^{\tau , \text{gen}}$. Both distributions are derived from a simulated sample of W(→*τν*)+jets events

As the muons in the muon control sample are selected to mimic a *τ*_h_, a correction is applied to emulate the probability to reconstruct and identify a *τ*_h_ lepton. The reconstruction and identification efficiency $\varepsilon_{\tau }^{\text{reco}}$ is parameterized as a function of the *p*_T_ of the *τ*_h_ candidate and as a function of the total number *N* of charged particles and photons in the isolation cone [Fig. 1(a)]. Corrections are also applied to account for the hadronic branching fraction ($f_{\tau}^{\text{bf(hadr)}}$) of a *τ* lepton. Except for the $f_{\tau}^{\text{bf(hadr)}}$ the values of the correction factors differ in each event. The corrections are combined to define an overall event weight, defined as:

2 $$ f^\text{corr}_\text{event} = \frac{P_{\mu}^{ \mathrm {W} } \times \varepsilon_{\tau} \times f_{\tau}^{\text{bf(hadr)}}}{\varepsilon _{\mu}^{\text{reco}} \times\varepsilon_{\mu}^{\text{iso}}}. $$

A *τ*_h_ response template is derived from simulated events. The response template is given by the ratio of the reconstructed energy of the *τ*_h_ to the true generator-level energy. The *τ*_h_ response depends on the transverse momentum of the generated *τ* lepton [Fig. 1(b)] and on the number of reconstructed primary vertices in the event. The muon *p*_T_ spectrum is smeared as a function of *p*_T_ and the number of primary vertices to mimic the *p*_T_ distribution of the *τ*_h_.

**Fig. 2:**
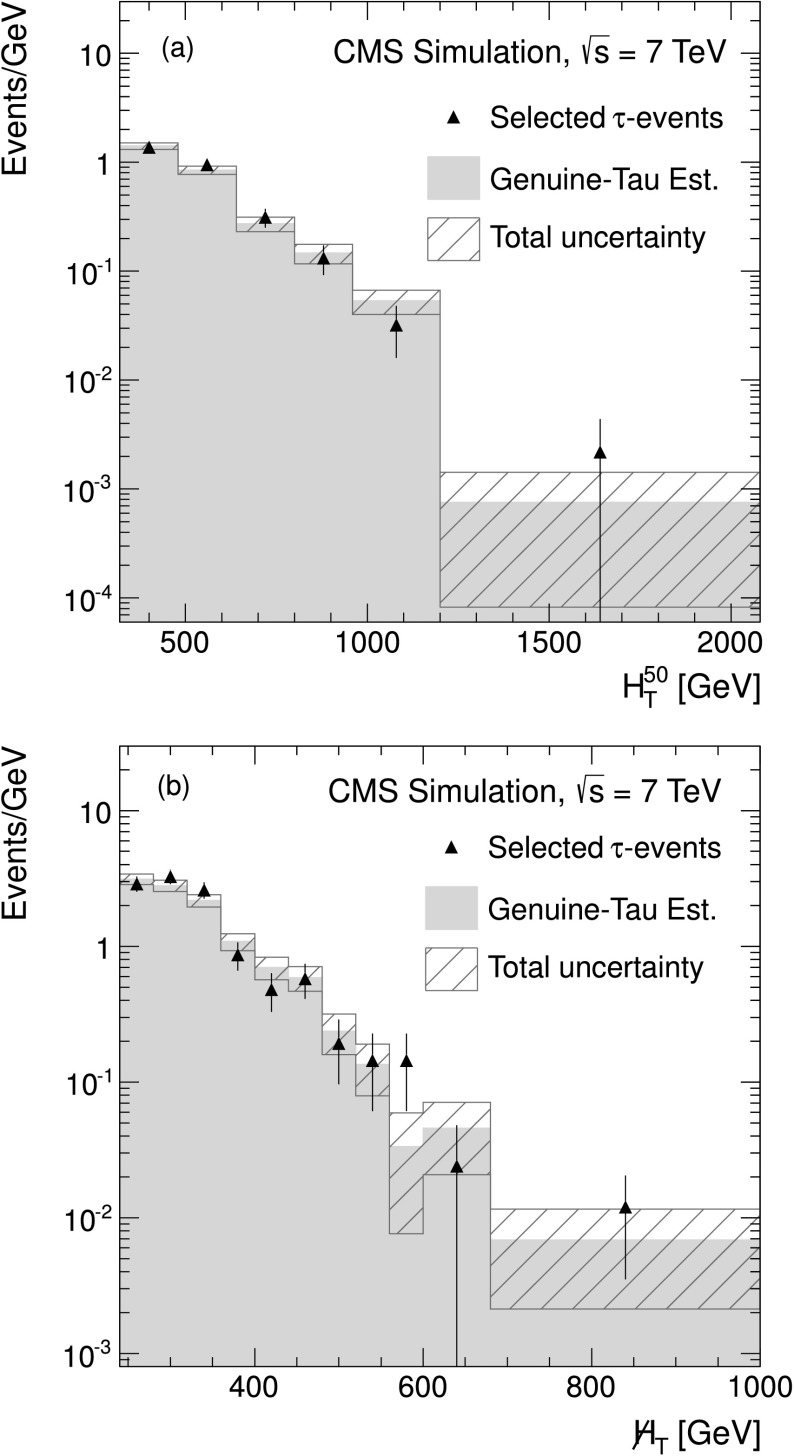
Distributions of (**a**) $H_{\mathrm{T}} ^{50}$ and (**b**)  for the genuine *τ*_h_ estimate in simulated W+jets events for the single-*τ*_h_ final state. The *black triangles* show the results for events that satisfy the baseline selection and that contain a reconstructed *τ*_h_ matched to the visible part of a generated, hadronically decaying *τ* lepton. The *filled green areas* show the prediction obtained from the simulated muon control sample. The *hatched areas* are the total uncertainty on the prediction (Color figure online)

Fully simulated W+jets events are used to verify the procedure. Figure 2 shows the $H_{\mathrm{T}} ^{50}$ and  distributions from simulated W+jets events for the single-*τ*_h_ final state. These events satisfy the baseline selection described in Sect. 5. The reconstructed *τ*_h_ is required to match a hadronically decaying generated tau lepton, to ensure that only the genuine tau background is addressed in this check. The event yield and distributions are compared with the prediction from the simulated muon control sample and agree within statistical uncertainties, thus verifying the closure of the method in MC simulation. Hence, the predicted $H_{\mathrm{T}} ^{50}$ and  distributions from the muon control sample can be taken to describe a *τ*_h_ sample within statistical uncertainties.

The muon control sample consists primarily of W+jets events, but also contains $\mathrm {t}\overline {\mathrm {t}} $ events in which one W boson decays into a muon while the other W boson decays either into an unidentified *τ* lepton or into a light lepton that is not reconstructed. Any isolated muons produced through the decay of b or c quarks can also contribute to the muon control sample. SM processes containing a Z boson or two W bosons can also contribute to the muon control sample if one of the two decay muons is not reconstructed.

The true event yields of each process as determined from simulation are summarized in Table 1 for the baseline and SR selections. For both selections the number of predicted events with a genuine *τ*_h_ lepton is seen to agree with the true number of events. The value of $\varepsilon_{\tau}^{\text{reco}}$ that is used to calculate the predicted rate is measured in a sample of W+jets and is different from the value that would be measured in a sample of $\mathrm {t}\overline{\mathrm {t}}$ events. This leads to an overestimation of the $\mathrm {t}\overline{\mathrm {t}}$ contribution. A systematic uncertainty is assigned to account for this overestimation.

**Table 1:** The selected and predicted background contributions for simulated events with a genuine *τ*_h_ passing the baseline and signal selection in the single-*τ*_h_ final state. The reconstructed *τ*_h_ is required to match the visible part of the generated, hadronically decaying *τ*-lepton. The predictions are derived from the muon control sample

*L*=4.98 fb^−1^	Baseline selection		Signal selection	
	Selected	Predicted	Selected	Predicted
W(→*ℓν*)+jets	452±30	441±21	28.9±7.5	34.9±5.9
$\mathrm {t}\overline{\mathrm {t}}$	60.6±3.7	63.2±2.1	1.6±0.6	2.9±0.4
Z(→*ℓℓ*)+ jets	10.9±2.1	8.4±1.3	0.8±0.6	0.4±0.3
W^+^W^−^	15.1±1.6	14.4±1.1	0.5±0.3	1.3±0.3
Sum	539±30	527±21	31.8±7.5	39.5±5.9

#### Estimate of the QCD multijet background in the single-*τ*_h_ final state

**Fig. 3:**
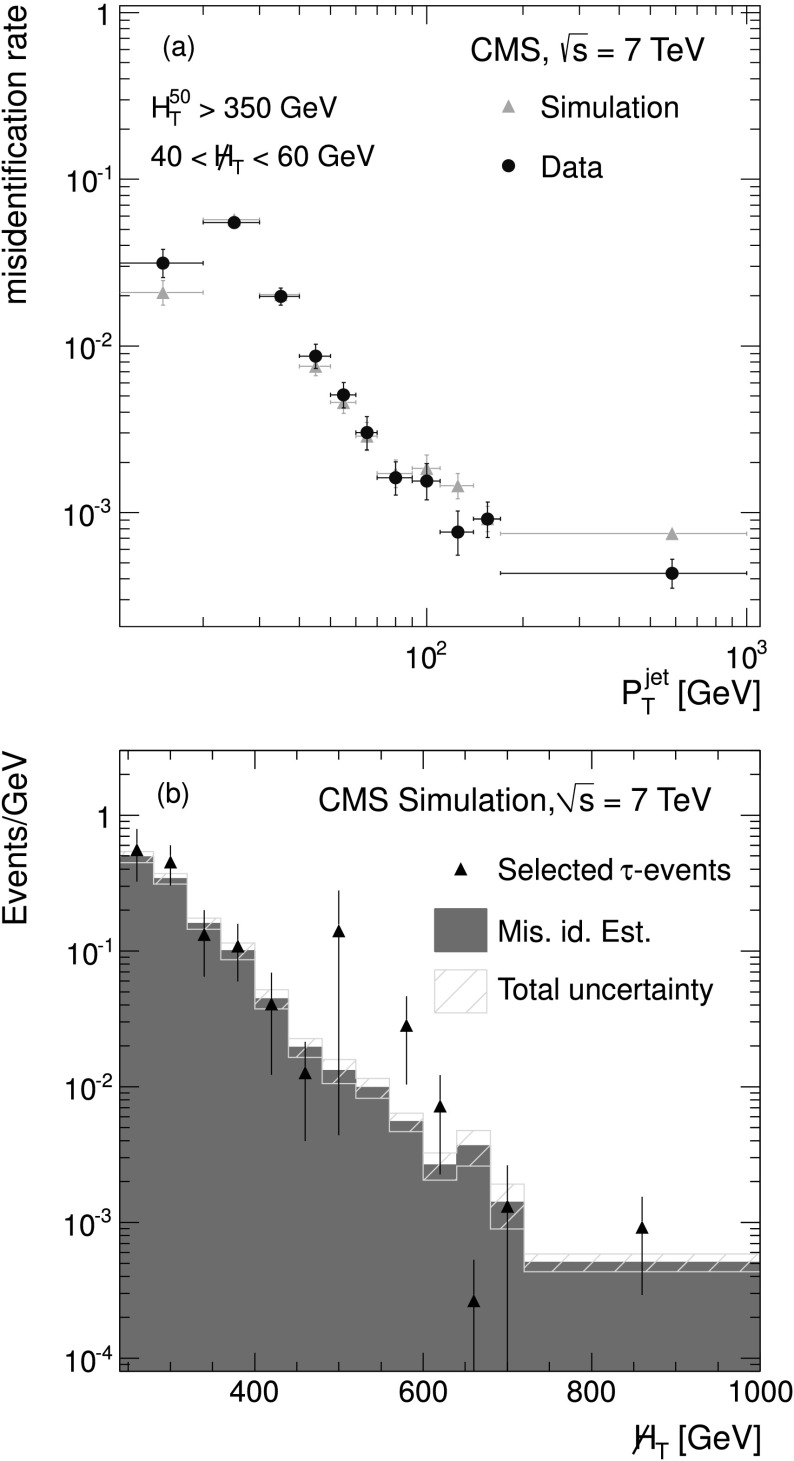
(**a**) The rate of jet misidentification as a *τ*_h_ lepton in simulation (*triangular symbols*) and data (*circular symbols*) as a function of $p_{\mathrm{T}} ^{\text{jet}}$ for events with $H_{\mathrm{T}} ^{50}>350 ~\text{GeV} $ and ; (**b**) The  distribution estimated in simulated events with $H_{\mathrm{T}} ^{50}>350 ~\text {GeV} $, where the *triangular symbols* represent events that pass the baseline selection, the *filled blue area* shows the predicted events, and the *hatched area* shows the total uncertainty on the prediction. These distributions correspond to the single-*τ*_h_ final state (Color figure online)

To estimate the background where a jet is misidentified as a *τ*_h_ lepton, a QCD-dominated control sample is obtained by selecting events with $H_{\mathrm{T}} ^{50} > 350 ~\text{GeV} $ and . The control sample is selected using a prescaled *H*_T_ trigger with criteria that lead to a sample where about 99 % of the events arise from QCD multijet production. The probability for a jet to be misidentified as a *τ*_h_ lepton is measured by determining the fraction of jets from the single-*τ*_h_ control sample that pass the *τ*_h_ identification criteria. Jets considered in the calculation of the misidentification rate satisfy the requirements *p*_T_>5 GeV and |*η*|<2.5. The misidentification rates *f*_*i*_ for each jet *i* depend on *η* and *p*_T_ and are used to determine an overall weight, which is applied to each event. The event weights are defined as:

3 $$ w^{\text{corr}}_{\text{event}} = 1 - \prod_i^n (1-f_i), $$

where *n* is the number of jets. The measured misidentification rates shown in Fig. 3(a) are applied to data events in the region with $H_{\mathrm{T}} ^{50}>350 ~\text {GeV} $ and with $H_{\mathrm{T}} ^{50}>600$ for two regions of :  and . These four regions are dominated by QCD multijet events. The results for data and simulation, as well as the predicted fraction of QCD multijet events, are shown in Table 2. The ratio of selected events over predicted events is statistically compatible with one and stable over the range of . Figure 3(b) shows the  distributions of predicted and selected events for simulated QCD multijet events with $H_{\mathrm {T}} ^{50}>350 ~\text{GeV} $. The two distributions agree over the whole range of .

**Table 2:** The percentage of QCD multijet events in the  binned samples for different QCD multijet dominated regions in the single-*τ*_h_ final state

$H_{\mathrm{T}} ^{50}>350 ~\text{GeV} $			
	60–80	80–100	>250
QCD fraction	97 %	93 %	6 %
selected/predicted (sim)	0.98±0.06	0.96±0.07	1.24±0.28
selected/predicted (data)	1.01±0.08	0.88±0.13	–
$H_{\mathrm{T}} ^{50}>600 ~\text{GeV} $			>400
QCD fraction	96 %	93 %	17 %
selected/predicted (sim)	0.94±0.09	0.85±0.09	2.43±1.45
selected/predicted (data)	1.14±0.26	0.97±0.37	–

### Estimate of backgrounds in the multiple-*τ*_h_ final state

The estimate of the SM background contributions to the SR sample for multiple-*τ*_h_ events is based on the number of observed events in CRs. The events in each CR are selected with similar selection requirements to those used in the SR, but are enriched with events from the background process in question. Correction factors and selection efficiencies are measured in those CRs and used to extrapolate to the SR. We use the observed jet multiplicity in each CR along with the measured rate at which a jet is misidentified as a *τ*_h_ to calculate the yield in the SR. The following equation is used to estimate each background contribution B:

4 $$ N_{\mathrm{B}}^\mathrm{SR} = N_{\mathrm{B}}^\mathrm{CR}\bigl[ \alpha _{\tau\tau}{\mathcal{P}} (0) + \alpha_{\tau j}{\mathcal{P}} (1) + \alpha_{jj}{\mathcal{P}} (2)\bigr], $$

where $N_{\mathrm{B}}^{\mathrm{SR}}$ is the predicted rate in the SR, $N_{\mathrm{B}}^{\mathrm{CR}}$ is the observed number of events in the CR, and *α*_*xy*_ is the correction factor for acceptance and efficiency for events in the CR with true physics objects “*x*” and “*y*”. Here the physics object can be a *τ*_h_ or a quark or gluon jet. Since the dominant SM backgrounds contribute to the SR when jets are misidentified as *τ*_h_ lepton, the background estimation strategy outlined in Eq. (4) relies on the determination of the event probability ${\mathcal{P}} (m)$ for at least “*m*” jets to be misidentified as a *τ*_h_, where ${\mathcal{P}} (m)$ is the product of three factors: (i) the probability *P*(*N*) for an event to contain *N* jets, (ii) the number of possible ways for exactly *n* jets to pass the *τ*_h_ identification criteria given *N* possible jets *C*(*N*,*n*)=*N*!/*n*!(*N*−*n*)!, and (iii) the probability *f* for a single jet to be misidentified as a *τ*_h_. The ${\mathcal{P}} (m)$ terms are given by:

5 $$ {\mathcal{P}} (m)=\sum_{N=m}^{\infty}P(N)\sum _{n=m}^{N}C(N,n)f^{n}(1-f)^{N-n}. $$

Equation (5) would be identical to Eq. (3) if used in the case of the single-*τ*_h_ final state. Equation (4) is used to estimate the $\mathrm {t}\overline{\mathrm {t}}$, W+jets, and Z+jets background contributions to the SR. The *P*(*N*) terms are determined from data using the jet multiplicity distribution in each CR, while the *f* terms are measured for each background process by determining the fraction of jets in each CR that pass the *τ*_h_ identification criteria. Since the QCD multijet contribution to the SR for the multiple-*τ*_h_ final state is negligible according to simulation, a data-to-MC scale factor is used to correct the QCD multijet prediction from simulation. In the sections that follow, the selections used to define high purity CRs are outlined and the correction factors *α*_*xy*_ used in Eq. (4) are defined. The fraction of events with two *τ*_h_ leptons is denoted *A*_*ττ*_, the fraction with one *τ*_h_ lepton and one jet misidentified as a *τ*_h_ lepton is denoted *A*_*τj*_, and the fraction with two jets misidentified as *τ*_h_ leptons is denoted *A*_*jj*_.

#### Estimate of the $\mathrm {t}\overline{\mathrm {t}}$ event background to the multiple-*τ*_h_ final state

**Fig. 4:**
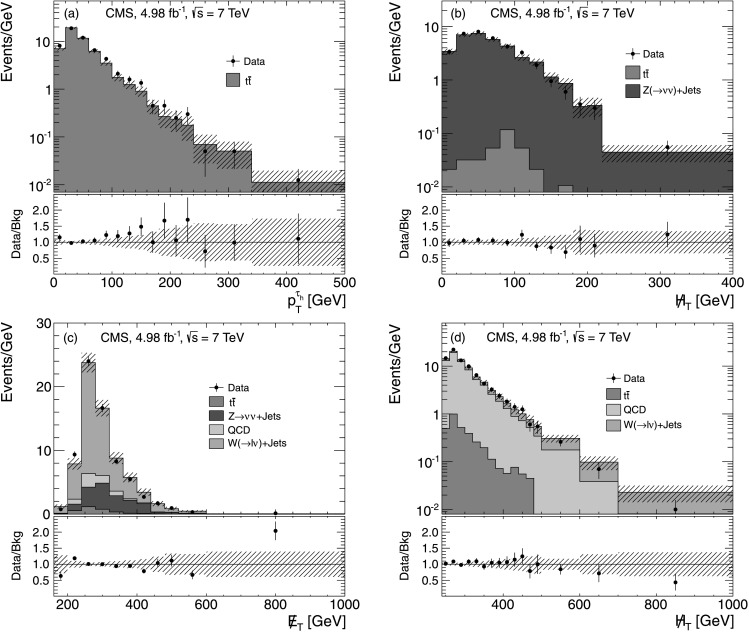
Data-to-MC comparison for the multiple-*τ*_h_ final state: (**a**) the *p*_T_ distribution of the *τ*_h_ candidate in the $\mathrm {t}\overline{\mathrm {t}}$ CR; (**b**)  distribution in the Z(→*μμ*)+jets CR; (**c**)  distribution in the W+jets CR; and (**d**)  distribution with the requirement . The *bottom panes* show the ratio between data and background while the hatched area depicts the total uncertainty on the MC

To estimate the contribution of $\mathrm {t}\overline{\mathrm {t}}$ events to the multiple-*τ*_h_ SR, a CR is selected by removing the *τ*_h_ isolation requirement and by requiring the presence of at least two b-quark jets (b jets), identified using the track-counting-high-efficiency (TCHE) algorithm at the medium working point [32]. Because QCD multijet, W+jets, Z(→*ττ*)+jets and Z(→*νν*)+jets events are unlikely to contain two b jets, this requirement provides a sample in which about 99 % of the events are $\mathrm {t}\overline{\mathrm {t}}$ events, according to simulation. Figure 4(a) shows the *p*_T_ distribution of *τ*_h_ leptons in the $\mathrm {t}\overline {\mathrm {t}}$ CR for data and simulation.

According to simulation, the fraction of events in the $\mathrm {t}\overline{\mathrm {t}}$ control sample that contains one genuine *τ*_h_ is *A*_*τj*_=0.166±0.011, while the fraction without a genuine *τ*_h_ is *A*_*jj*_=0.834±0.025. The genuine *τ*_h_ *τ*_h_ contribution is negligible (*A*_*ττ*_∼0) according to simulation. Incomplete knowledge of the genuine *τ*_h_ *τ*_h_ contribution is included as a source of systematic uncertainty in the $\mathrm {t}\overline{\mathrm {t}}$ background prediction. Therefore, *α*_*τj*_ in Eq. (4) is given by $A_{\tau j}{\varepsilon_{\tau}^{\text{iso}}}/{P(\text{2 $\mathrm {b}$\ jets})}$, where $\varepsilon_{\tau}^{\text{iso}}$ is the probability for a *τ*_h_ lepton to pass the isolation requirement, while *α*_*jj*_ is given by *A*_*jj*_/*P*(2 b jets). The probability $P(\text{2 $\mathrm {b}$\ jets})$ to identify two or more b jets is determined by the b jet identification efficiency factor [32]. The number of $\mathrm {t}\overline{\mathrm {t}}$ events in the SR is calculated as:

6 $$ N_{ \mathrm {t}\overline{\mathrm {t}} }^\mathrm{SR} = \frac{N^\mathrm{CR}_{t \overline{t}}}{P(\text{2 $\mathrm {b}$\ jets})}\bigl[ A_{\tau j} \varepsilon_{\tau}^{\text{iso}}{\mathcal{P}} (1) + A_{j j}{ \mathcal{P}} (2) \bigr]. $$

The probability for a jet in a $\mathrm {t}\overline {\mathrm {t}}$ event to be misidentified as a *τ*_h_ lepton has an average measured value of *f*=0.022±0.004. Cross checks are made to validate the use of the b-jet identification efficiency as measured in Ref. [32] for this analysis. The estimated $\mathrm {t}\overline{\mathrm {t}}$ contribution in the SR is determined to be $N_{ \mathrm {t}\overline{\mathrm {t}} }^{\mathrm{SR}} = 2.03 \pm0.36$.

#### Estimate of the Z(→*νν*)+jets event background to the multiple-*τ*_h_ final state

The contribution of Z(→*νν*)+jets events to the multiple-*τ*_h_ SR is evaluated by selecting a sample of Z(→*μμ*)+jets events and treating the muons as neutrinos. The sample is collected using a trigger designed to select a muon and a *τ*_h_. Jet selection criteria similar to those used for the SR sample are imposed. In addition, we require two muons passing the criteria outlined in Sect. 3. The control sample has a purity of about 99 % as estimated from simulation. The  distribution for events in this CR is shown in Fig. 4(b). The Z(→*νν*)+jets background is estimated by interpreting the *p*_T_ of the pair of muons as . In order to predict the Z(→*νν*)+jets rate in the SR, the Z(→*μμ*)+jets sample is corrected for the ratio of the branching fractions *R*=*B*(Z→*νν*)/*B*(Z→*μμ*), for trigger efficiencies, for the geometric acceptance *A*_*μ*_ as measured from simulation, and for the reconstruction efficiency $\varepsilon_{\mu}^{\text{reco}}$ as measured from data. Therefore, *α*_*jj*_ in Eq. (4) is given by: 7


Since there is no prompt production of a genuine *τ*_h_ in the Z(→*μμ*)+jets sample, *α*_*τj*_=0 and *α*_*ττ*_=0. The Z(→*νν*)+jets contribution to the SR is calculated as: 8 where  is the  trigger efficiency and $\varepsilon^{\text{Trigger}}_{\mu\tau}$ the *μτ*_h_ trigger efficiency. The efficiency for the  signal selection () is determined by calculating the fraction of the observed events in the CR that have . The muon identification efficiency *ε*_*μ*_ is measured using a “tag-and-probe” method. The probability for a jet to be misidentified as a *τ*_h_ lepton has a measured value of *f*=0.016±0.002. The estimated Z(→*νν*)+jets contribution to the SR is determined to be $N_{ \mathrm {Z} (\to\nu\nu )}^{\mathrm{SR}} = 0.03 \pm0.02$.

#### Estimate of the Z(→*ττ*)+jets event background to the multiple-*τ*_h_ final state

The contribution from Z→*ττ* events is determined with the Z(→*μμ*)+jets CR sample used to estimate the background from Z(→*νν*)+jets, with the muons treated as *τ*_h_ leptons. The *α*_*xy*_ factors are more difficult to estimate for Z→*ττ* events since there are several ways in which Z→*ττ* events can contribute to the SR: (i) both *τ*_h_ leptons pass the kinematic acceptance and identification criteria; (ii) both *τ*_h_ leptons pass the kinematic acceptance criteria, but only one passes the identification criteria; (iii) one *τ*_h_ fails the kinematic acceptance criteria, while the other *τ*_h_ passes both the kinematic acceptance and identification criteria; or (iv) both *τ*_h_ leptons fail the kinematic acceptance criteria. The Z(→*ττ*)+jets contribution to the SR is calculated as:

9 $$ \begin{aligned}[b] N_{ \mathrm {Z} \to\tau\tau}^\mathrm{SR} &= N^\mathrm{CR}_{ \mathrm {Z} \to\mu\mu}R \biggl[ \frac{A_{\tau}^{2}\varepsilon_{\tau}^{2}}{A_{\mu}^{2}\varepsilon _{\mu}^{\text{reco 2}}} + \frac{2A_{\tau}^{2}\varepsilon_{\tau}(1-\varepsilon_{\tau })}{A_{\mu}^{2}\varepsilon_{\mu}^{\text{reco 2}}}{\mathcal{P}} (1) \\ &\quad {}+\frac{2A_{\tau}(1-A_{\tau})\varepsilon_{\tau}}{A_{\mu }^{2}\varepsilon_{\mu}^{\text{reco 2}}}{\mathcal{P}} (1) + \frac{(1-A_{\tau})^{2}}{A_{\mu}^{2}\varepsilon_{\mu}^{\text{reco 2}}}{\mathcal{P}} (2) \biggr], \end{aligned} $$

where *R* is given by: 10
*A*_*τ*_ is the *τ*_h_ acceptance, *ε*_*τ*_ is the *τ*_h_ identification efficiency in this control sample, and *f*=0.016±0.002. The estimated Z (→*ττ*) + jets contribution to the SR is determined to be $N_{{ \mathrm {Z} } (\to\tau\tau )}^{\mathrm{SR}} = 0.21 \pm0.13$.

#### Estimate of the W + jets event background to the multiple-*τ*_h_ final state

To select the W+jets CR, the *τ*_h_ isolation requirement, which discriminates between a *τ*_h_ lepton and other jets, is removed from the SR selection requirements. However, the lack of the *τ*_h_ isolation requirement increases the contribution from other backgrounds as most of the backgrounds arise because jets are misidentified as a *τ*_h_ lepton. To minimize the contribution from $\mathrm {t}\overline{\mathrm {t}}$ production, events are required to have no jets identified as a b jet. This requirement reduces the contamination from $\mathrm {t}\overline{\mathrm {t}}$ events to around 5 %. The purity of the W+jets CR is approximately 65 %. Figure 4(c) shows the  distribution, defined as the magnitude of the negative of the vector sum of the transverse momentum of all PF objects in the event, for events in the W+jets CR. The contributions of QCD multijet, $\mathrm {t}\overline{\mathrm {t}}$, and Z(→*νν*)+jets events are subtracted in order to determine the number of W+jets events in the CR. The predicted rates for QCD multijet, $\mathrm {t}\overline{\mathrm {t}}$, and Z(→*νν*)+jets events are determined by extrapolating from their corresponding CRs. Since there is no genuine multiple-*τ*_h_ production in W+jets, *α*_*ττ*_=0. According to simulation, the fraction of events in the CR with one genuine *τ*_h_ is *A*_*τj*_=0.149±0.016, while the fraction of events without a genuine *τ*_h_ is *A*_*jj*_=0.851±0.038. Therefore, *α*_*τj*_ in Eq. (4) is given by $A_{\tau j}{\varepsilon_{\tau}^{\text{iso}}}/{P(\text{0 $\mathrm {b}$\ jets})}$, where $\varepsilon_{\tau}^{\text{iso}}$ is the probability for a *τ*_h_ to pass the isolation requirement and $P(\text{0 $\mathrm {b}$\ jets})$ is the probability to not have any light-quark or gluon jet misidentified as a b jet. Similarly, *α*_*jj*_ is given by ${A_{jj}}/{P(\text{0 $\mathrm {b}$\ jets})}$. The contribution of W+jets events to the SR is then calculated as:

11 $$ N_{W+\text{jets}}^\mathrm{SR} = \frac{N^{\text{After subtraction}}_{ \mathrm{W} +\textrm {jets}}}{P(\text{0 $\mathrm {b}$\ jets})} \bigl[ A_{\tau j} \varepsilon _{\tau}^{\text {iso}}{\mathcal{P}} (1) + A_{jj}{ \mathcal{P}} (2) \bigr]. $$

The average rate at which jets are misidentified as a *τ*_h_ lepton is measured to be 0.019±0.001. The rate *f*_b_ at which light-quark jets or gluon jets are misidentified as a b jet is used to determine $P(\text{0 $\mathrm {b}$\ jets})$. The estimated W+jets contribution to the SR is determined to be $N_{ \mathrm{W} + \textrm{jets}}^{\mathrm{SR}} = 5.20 \pm0.63$.

#### Estimate of the QCD multijet event background to the multiple-*τ*_h_ final state

QCD multijet events contribute to the multiple-*τ*_h_ SR when mismeasurements of jet energies lead to large values of  and when jets are misidentified as *τ*_h_ candidates. By removing the *τ*_h_ isolation criteria and inverting the  requirement, a QCD CR sample with about 99 % purity is obtained. Figure 4(d) shows the expected and observed  distributions for this sample. A scale factor is obtained from this CR and used to correct the signal prediction for QCD multijet events in simulation. The estimated contribution to the SR from QCD multijet events is determined to be $N_{\mathrm{QCD}}^{\mathrm{SR}} = 0.02 \pm0.02$.

## Systematic uncertainties

Systematic uncertainties are taken into account for both signal and background events and are described separately. Both the signal and background are affected by the systematic uncertainty in the identification of the *τ*_h_ candidate. The systematic uncertainty for *τ*_h_ identification is obtained using a Z→*ττ* enhanced region and by correcting this cross section by that measured for $\mathrm {Z} \to{\rm ee}$ and Z→*μμ* events. This uncertainty is validated on a control sample of Z→*ττ* events. The level of agreement between data and simulation is found to be at the level of 7 %. Further validation of the performance of *τ*_h_ identification in a SUSY-like environment is performed by selecting a W(→*τν*→*τ*_h_ *νν*) +jets CR with large hadronic activity (*H*_T_) and large transverse momentum imbalance (). The level of agreement between the predicted rate for W(→*τν*→*τ*_h_ *νν*) events and the observed number of events is within 7 % and is determined as a function of *H*_T_ and .

### Systematic uncertainties on background events

The principal sources of systematic uncertainty on the background predictions arise from the correction factors, the finite number of events in the CRs, the measured rates at which jets are misidentified as a *τ*_h_ lepton, and the level of agreement between the observed and predicted numbers of events in CRs.

The contributions to the uncertainties on the correction factors are different for each background category. The dominant effect is due to the uncertainty in the *τ*_h_ identification efficiency. In the multiple-*τ*_h_ final state, uncertainties in the jet-energy scale (JES) [33] and the *τ*_h_-energy scale (TES) [34] are used to evaluate how changes in *H*_T_, , and jet kinematics affect the correction factors. The systematic uncertainty on the correction factors due to the JES and TES is at most ∼3 % for all backgrounds. Smaller contributions to the uncertainties in the correction factors arise from the muon reconstruction and isolation efficiency (<1 %), the uncertainty in the branching fractions (≪1 %), and the uncertainties in trigger efficiency (1 %).

The systematic uncertainties on the measured rates for jet misidentification as a *τ*_h_ lepton are dominated by the size of the jet sample used to measure these rates and range from 2 % for the single-*τ*_h_ final state to 5.6–10 % for the multiple-*τ*_h_ final state. The level of agreement between the observed and predicted number of events in MC studies of the CRs is used to assign an additional systematic uncertainty and ranges from 2 % for the single-*τ*_h_ final state to 3 % for the multiple-*τ*_h_ final state. Finally, the systematic uncertainty arising from statistical uncertainties on the number of events in the CRs ranges from 2–5 % for the multiple-*τ*_h_ final state to 3–10 % for the single-*τ*_h_ final state.

### Systematic uncertainties on signal events

The main sources of systematic uncertainties in the SR are due to trigger efficiencies, identification efficiencies, the energy, and momentum scales, the luminosity measurement and PDFs. The uncertainty on the luminosity measurement is 2.2 % [35]. Systematic uncertainties on the  triggers (2.5 %) are measured using a sample in which around 99 % of the events are $\mathrm {t}\overline{\mathrm {t}}$ events, which have a similar topology to events in the SR samples. The systematic uncertainties on the TES and JES (3.0 %) yield an uncertainty on the signal acceptance of 2.3 %. The uncertainty on the  scale depends on the uncertainty of the JES (2–5 % depending on the *η* and *p*_T_ values of the jet) and on the unclustered energy scale (10 %). Unclustered energy is defined as the energy found “outside” any reconstructed lepton or jet with *p*_T_>10 GeV. The unclustered energy scale uncertainty has a negligible systematic uncertainty on the signal acceptance. The systematic uncertainty due to imprecise knowledge of the PDFs (11 %) is determined by comparing the CTEQ6.6L [36], MSTW 2008 NLO [37], and NNPDF2.1 [38] PDFs with the default PDF [39]. The systematic uncertainty due to the imprecise modeling of the initial-state and final-state radiation [40] is negligible (≪1 %). The systematic uncertainties associated with event pileup are also negligible. Uncertainties on the theoretical cross sections are evaluated by varying the PDFs and by changing the renormalization and factorization scales by a factor of two [26–31].

## Results

**Fig. 5:**
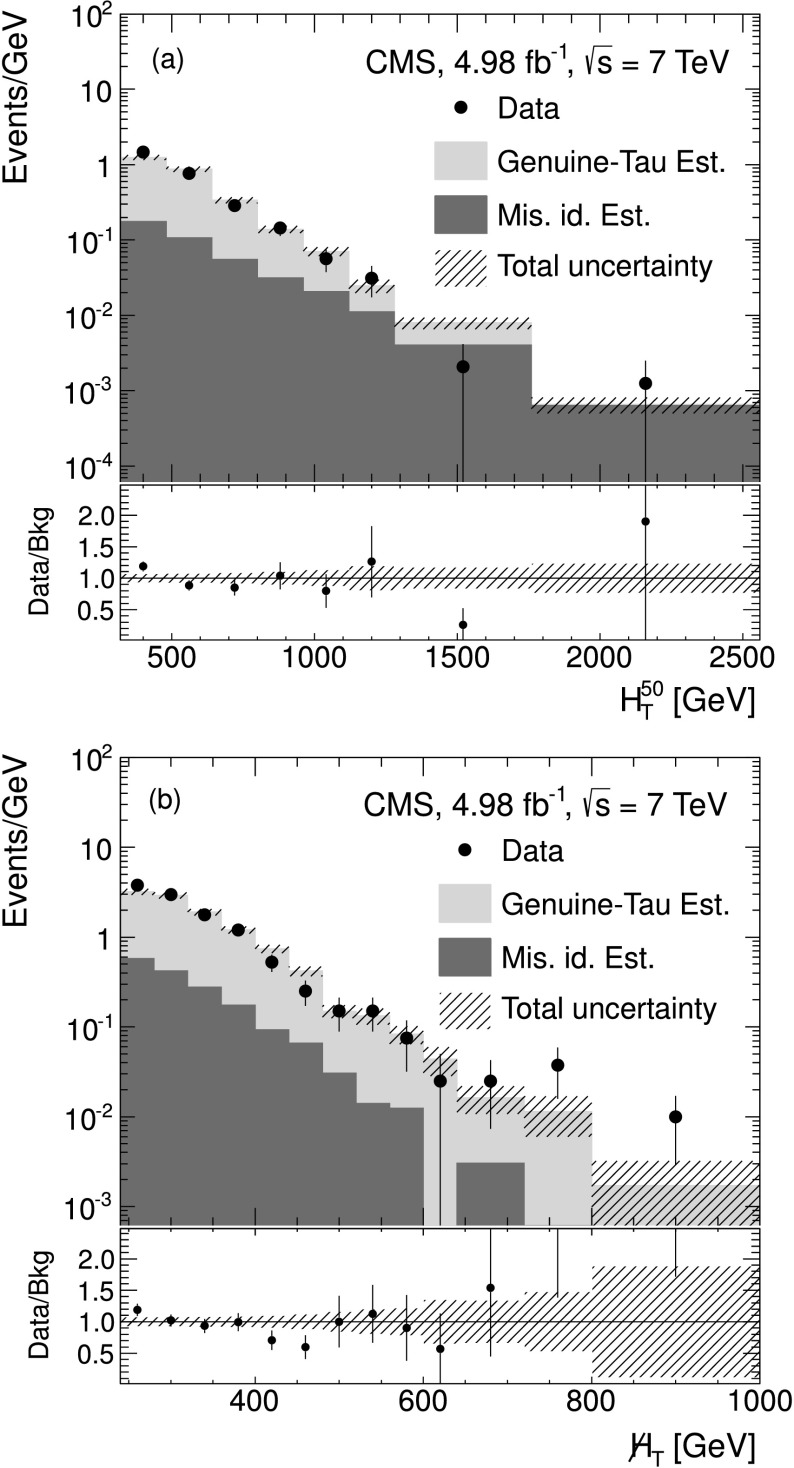
Distributions of (**a**) $H_{\mathrm{T}} ^{50}$, and (**b**)  for the single-*τ*_h_ final state. The *points* with errors represent data that satisfy the baseline selection while the filled *green* (*light*) and filled *blue* (*dark*) areas shows the predicted backgrounds due to events containing a genuine *τ*_h_ and a misidentified *τ*_h_, respectively. The *hatched area* shows the total uncertainty on the prediction (Color figure online)

For the single-*τ*_h_ final state, the number of background events containing a genuine *τ*_h_, as well as the number of background events containing a misidentified *τ*_h_, are estimated with data. The results for the baseline and the full selection are listed in Table 3. Figure 5 shows the $H_{\mathrm {T}} ^{50}$ and  distributions of data and the different background predictions. The observed number of events in data is in agreement with the SM predictions.

**Table 3:** Number of data and estimated background events with statistical and systematic uncertainties, respectively, in the single-*τ*_h_ final state

Process	Baseline	Signal region
Fake-*τ*_h_	67±2±19	3.4±0.4±1.0
Real-*τ*_h_	367±10±27	25.9±2.5±2.3
Estimated ∑*SM*	434±10±33	29.3±2.6±2.5
Data	444	28

**Fig. 6:**
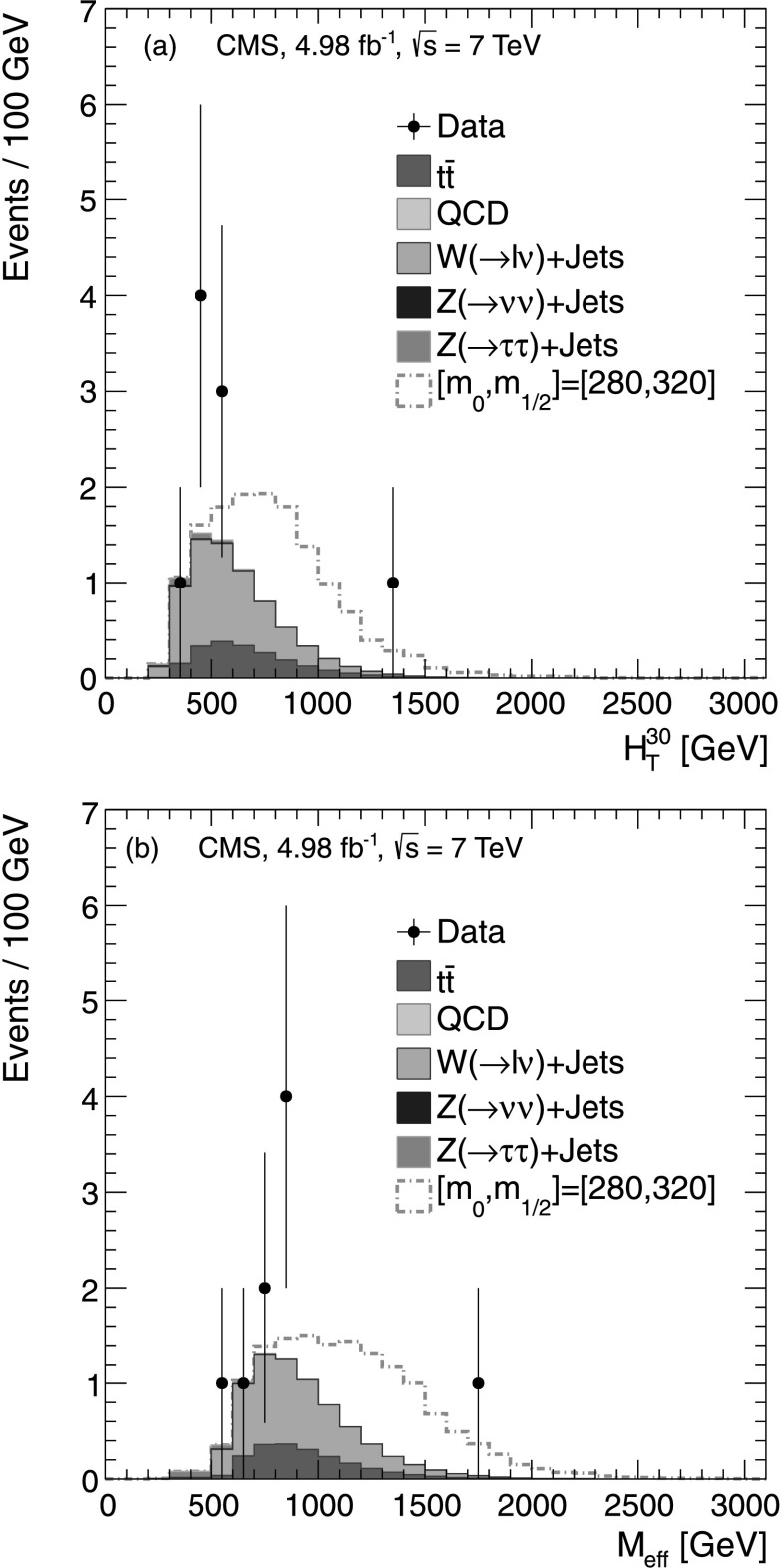
Stacked distributions of (**a**) $H_{\mathrm {T}} ^{30}$, and (**b**) *M*_eff_ in the SR for the multiple-*τ*_h_ final state. The background distributions are taken from MC events that are normalized to the predictions based on data over the *full region*. The shapes obtained from MC simulation are used for illustrative purposes only

The largest sources of background for the multiple-*τ*_h_ final state are from $\mathrm {t}\overline {\mathrm {t}}$ and W+jets events. A counting experiment is performed and the background predictions from data are compared with the observed number of events. Table 4 lists these background predictions and the observed number of events in the SR. Figure 6 shows the $H_{\mathrm{T}} ^{30}$ as well as the *M*_eff_ distributions in the SR, where *M*_eff_ is the sum . The background distributions in Fig. 6 are taken from simulation and normalized over the full spectrum. The estimated number of events due to the SM background processes is in agreement with the number of observed events in the SR.

**Table 4:** Number of data and estimated background events with statistical and systematic uncertainties, respectively, in the multiple-*τ*_h_ final state

Process	Signal region
QCD multijet events	0.02±0.02±0.17
W+jets	5.20±0.63±0.62
$\mathrm {t}\overline{\mathrm {t}} $	2.03±0.36±0.34
Z(→*ττ*)+jets	0.21±0.13±0.17
Z(→*νν*)+jets	0.03±0.02±0.50
Estimated ∑*SM*	7.49±0.74±0.90
Data	9

## Limits on new physics

**Fig. 7:**
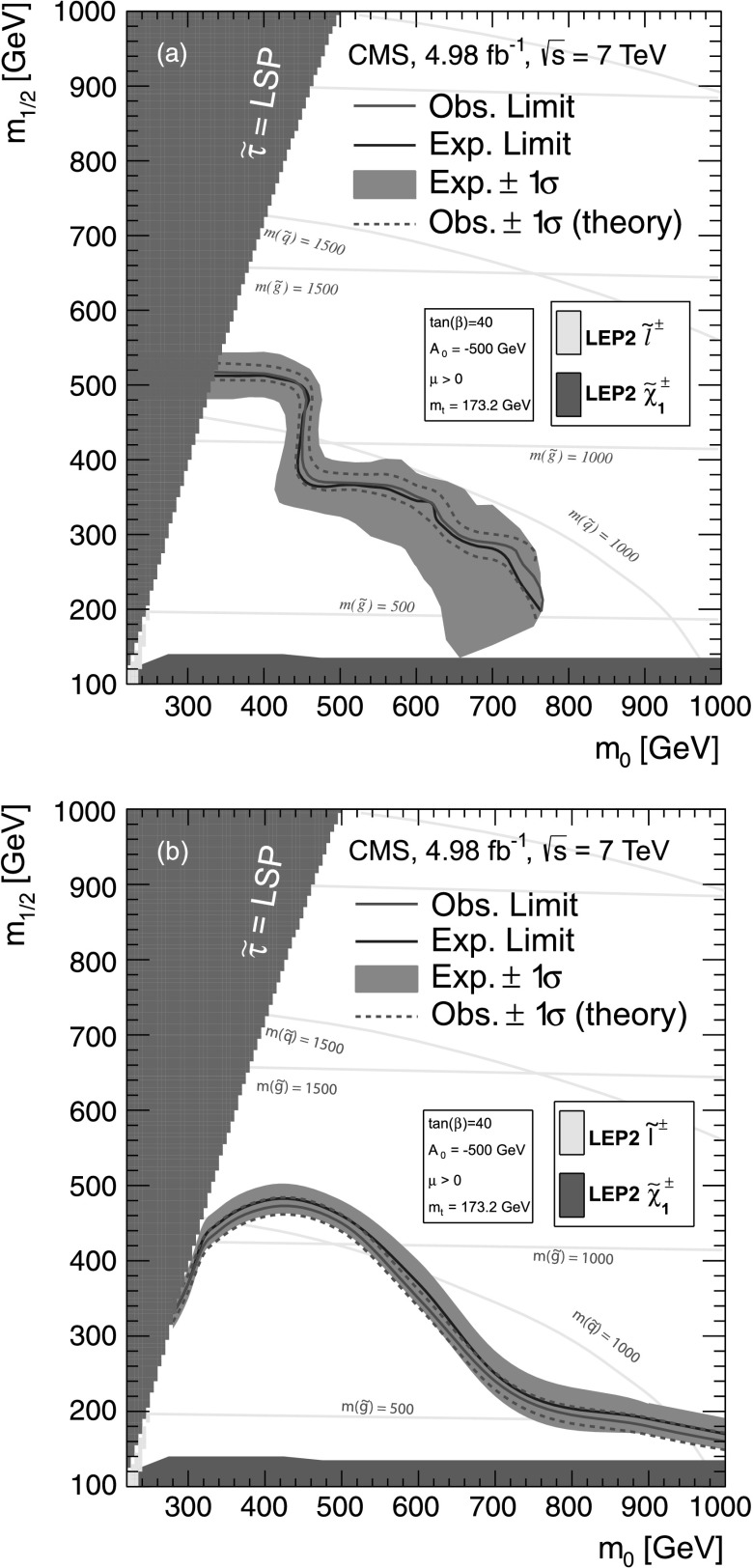
95 % CL exclusion limits in the CMSSM plane at tan*β*=40 for: (**a**) Single-*τ*_h_ final state, and (**b**) multiple-*τ*_h_ final state. In the figures shown, the *solid red line* (Obs. Limit) denotes the experimental limit while the *dotted red lines* (Obs. ±*σ* (theory)) represent the uncertainty on the experimental limit due to uncertainties on the theoretical cross sections. The *blue band* (Exp. ±*σ*) represents the expected uncertainties. The contours of constant squark and gluino mass are in units of  GeV (Color figure online)

The observed numbers of events in the single-*τ*_h_ and multiple-*τ*_h_ final states do not reveal any evidence of physics beyond the standard model. Exclusion limits are set using the CL_s_ [41] criterion in the context of the CMSSM [42]. The CMSSM parameter space with tan*β*=40, *A*_0_=−500 GeV, *μ*>0, and *M*_t_=173.2 GeV is chosen as a possible scenario with a light $\widetilde{ \tau } $ and a value of Δ*M*≤20 GeV. The excluded regions are shown for the single-*τ*_h_ and multiple-*τ*_h_ final states in Figs. 7(a) and 7(b), respectively. The limits are set using a simple counting experiment. Systematic uncertainties are treated as nuisance parameters and marginalized, and contamination from signal events in the control samples is taken into account. In the CLs method, both the background-only as well as the signal + background hypothesis are used to derive the confidence levels CL_s_ and the resulting limits and the uncertainty bands on the exclusion contours. In the case of very small values of Δ*M*(∼5 GeV), the lower-energy *τ*_h_ cannot be effectively detected and only the energetic *τ*_h_ from the decay of the neutralino can be observed. The search for new physics with a single *τ* lepton has a better sensitivity in this case. The single-*τ*_h_ and multiple-*τ*_h_ topologies thus have complementary sensitivity and together provide coverage for models with a wide range of Δ*M* values.

Using the limits set by the single-*τ*_h_ analysis, a common gaugino mass *m*_1/2_ of <495 GeV is excluded at 95 % Confidence Level (CL) for a common scalar mass *m*_0_ of <440 GeV. For the multiple-*τ*_h_ analysis, *m*_1/2_<465 GeV is excluded at 95 % CL for *m*_0_=440 GeV. A gluino with mass <1.15 TeV is excluded at 95 % CL for *m*_0_<440 GeV. It can be noted that the single-*τ*_h_ analysis shows better sensitivity for small values of Δ*M*, which is near the boundary of $\widetilde { \tau } = \mathrm{LSP}$.

**Fig. 8:**
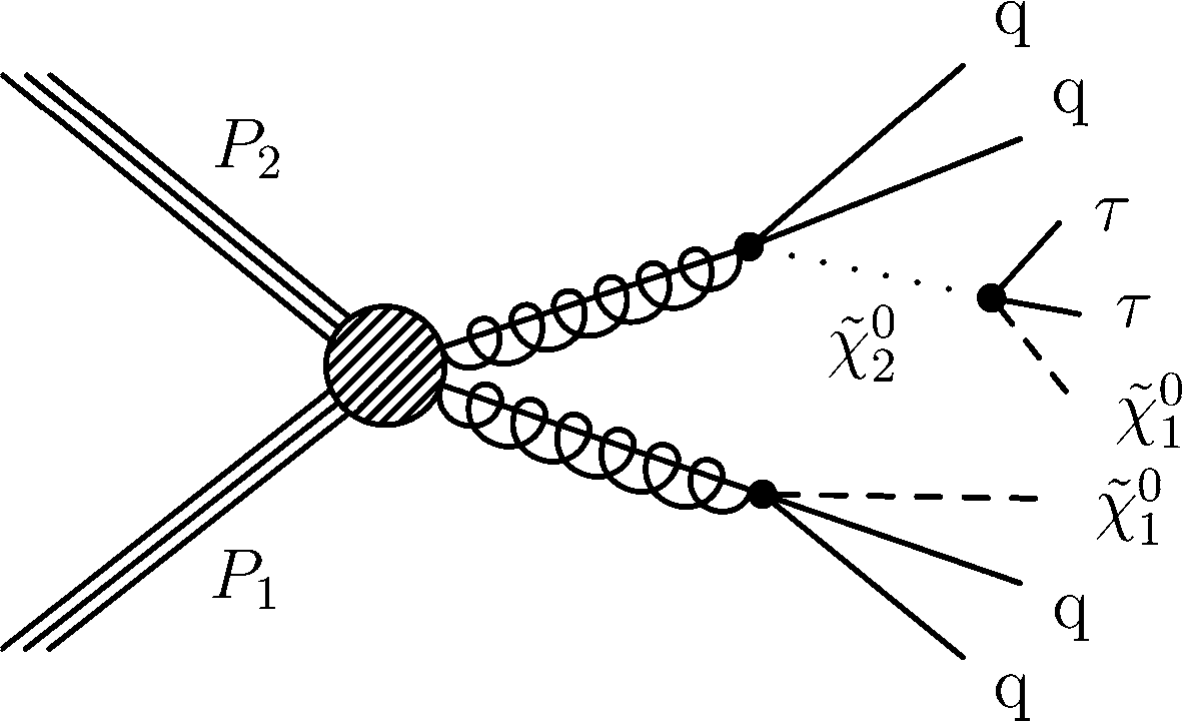
Diagram for the T3tauh SMS model

**Fig. 9:**
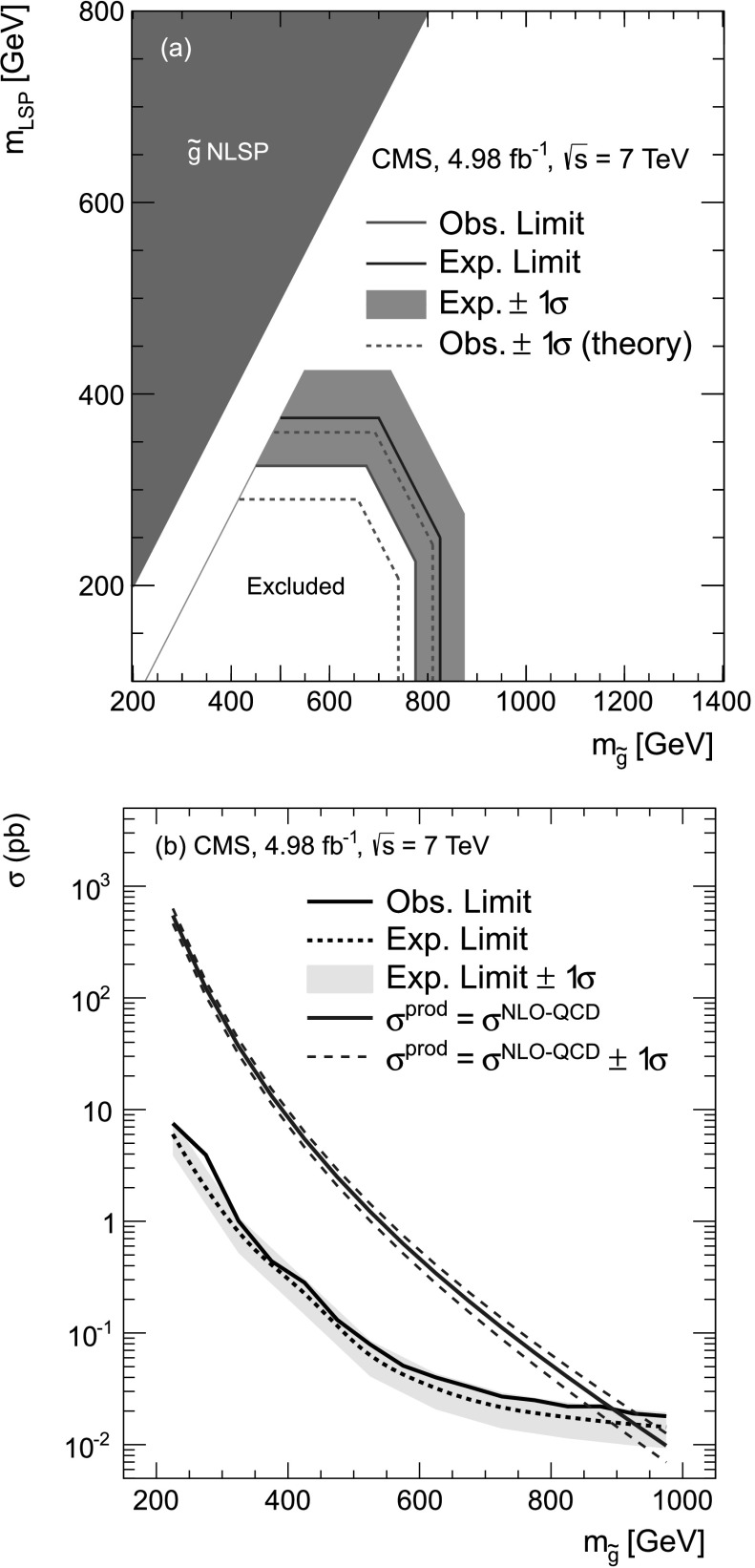
Exclusion limits for the multiple-*τ*_h_ final state: (**a**) 95 % CL exclusion region obtained for the T3tauh model, where the *solid red line* represents the limits on the mass of the gluino and the LSP; (**b**) 95 % CL cross section upper limits as a function of gluino mass in the GMSB scenario. In this figure *σ* ^prod^ represents the cross section for the production of a pair of gluinos with subsequent decay into *τ* lepton pairs at a 100 % branching fraction (Color figure online)

The results for the multiple-*τ*_h_ final states are also interpreted in the context of SMS [13]. The *ττ* SMS scenario (T3tauh) is studied where gluinos are produced in pairs and subsequently decay to *τ* lepton pairs and an LSP via a neutralino ($\widetilde{\mathrm{g}} \to \mathrm {q} \overline{\mathrm {q}} \widetilde {\chi}^{0}_{2} $; $\widetilde{\chi }^{0}_{2} \to\tau\overline{\tau} \to\tau\tau \widetilde{\chi}^{0}_{1} $). The diagram for the T3tauh model is given in Fig. 8. A gluino mass of <740 GeV is excluded at 95 % CL for LSP masses up to 205 GeV (here, the mass of $\widetilde{\chi}^{0}_{2} $ is the average of the masses of the gluino and the LSP). Figure 9(a) shows the 95 % CL exclusion region obtained for T3tauh. The limits on the mass of the gluino and LSP are shown with a solid red line.

In the simplified GMSB scenario, the $\widetilde{ \tau } $ is the NLSP and decays to a *τ* lepton and a gravitino $\widetilde{ \mathrm {G} } $, with a mass of the order of ∼ keV [43–45] ($\widetilde{\chi}^{0}_{2} \to\tau \widetilde{ \tau } \to\tau\tau \widetilde{ \mathrm {G} } $). The topology for this simplified GMSB scenario is similar to that of T3tauh except for the assumption that both the gluinos decay to *τ*-lepton pairs with a branching fraction of 100 %. Therefore, the results are also interpreted in the simplified GMSB scenario using the T3tauh scenario. The signal acceptance is corrected to account for the final state containing up to four *τ* leptons. A gluino with mass <860 GeV is excluded at 95 % CL. Figure 9(b) shows the exclusion limits for the simplified GMSB scenario as a function of the gluino mass.

Since the SMS topologies considered in this paper are characterized by two *τ* leptons in the final state, we do not present SMS limits for the single-*τ*_h_ final state.

## Summary

A search for physics beyond the standard model with one or more hadronically decaying *τ* leptons, highly energetic jets, and large transverse momentum imbalance in the final state is presented. The data sample corresponds to an integrated luminosity of 4.98±0.11 fb^−1^ of pp collisions at $\sqrt{s}=7 ~\text{TeV} $ collected with the CMS detector. The final number of events selected in data is consistent with the predictions for standard model processes. We set upper limits on the cross sections for the CMSSM, GMSB, and SMS scenarios. Within the CMSSM framework at tan*β*=40, a gaugino mass *m*_1/2_<495 GeV is excluded at 95 % CL for scalar masses *m*_0_<440 GeV. This result sets a lower limit on the mass of the gluino at 1.15 TeV with 95 % CL in this region. In the multiple-*τ*_h_ final state, a gluino with a mass less than 740 GeV is excluded for the T3tauh simplified model while a gluino with a mass less than 860 GeV is excluded for the simplified GMSB scenario at 95 % CL.
